# Supplementary material for: Latrine utilization and associated factors among districts implementing and not-implementing community-led total sanitation and hygiene in East Wollega, Western Ethiopia: A comparative cross-sectional study
Source: PLoS One. 2023 Jul 13;18(7):e0288444. doi: 10.1371/journal.pone.0288444 (PMC10343159; doi:10.1371/journal.pone.0288444)
Supplement: S1 Checklist — (DOCX) [file pone.0288444.s001.docx]

STROBE Statement—checklist of items that should be included in reports of observational studies

|  | Item No. | Recommendation | Page  No. | Relevant text from manuscript |
| --- | --- | --- | --- | --- |
| **Title and abstract** | 1 | (*a*) Indicate the study’s design with a commonly used term in the title or the abstract | 1-2 | **a comparative cross-sectional study design** |
|  |  | (*b*) Provide in the abstract an informative and balanced summary of what was done and what was found | 2 | The prevalence and associated factors of latrine utilization among households in community-led total sanitation and hygiene implemented and non-implemented Districts were determined. Accordingly, the prevalence of latrine utilization in CLTSH implemented district was found to be 53.7% [95%CI:47%, 60.3%] while it was 51.7% [95%CI:45.1%, 58.3%] in CLTSH non-implemented district. Overall, 52.7% [(95%CI:48%, 57.3%] of the households utilized their latrine. |
| Introduction | | | |  |
| Background/rationale | 2 | Explain the scientific background and rationale for the investigation being reported | 3-5 | So far conducted studies identified that latrine utilization is affected by socio-demographic and economic, behavioral, and environmental factors. To the authors’ knowledge, there is a paucity of the study to compare the prevalence of latrine utilization among villages in CLTSH-implemented and non-implemented districts. Besides, the status of CLTSH implementation and challenges are not well addressed in many of the previous studies. |
| Objectives | 3 | State specific objectives, including any prespecified hypotheses | 5 | This study was intended to compare the prevalence of latrine utilization and associated factors among the CLTSH-implemented and non-implemented districts of East Wollega, Western Ethiopia. |
| Methods | | | |  |
| Study design | 4 | Present key elements of study design early in the paper | 5 | A comparative community-based cross-sectional study design mixed with a qualitative method was employed. |
| Setting | 5 | Describe the setting, locations, and relevant dates, including periods of recruitment, exposure, follow-up, and data collection | 5 | The study was conducted in the East Wollega Zone, Oromia Regional State of Ethiopia from July 1 to 30, 2022. East Wollega is located 331 km far from Addis Ababa, the capital city of Ethiopia. |
| Participants | 6 | (*a*) *Cohort study*—Give the eligibility criteria, and the sources and methods of selection of participants. Describe methods of follow-up  *Case-control study*—Give the eligibility criteria, and the sources and methods of case ascertainment and control selection. Give the rationale for the choice of cases and controls  *Cross-sectional study*—Give the eligibility criteria, and the sources and methods of selection of participants | 5 and 6 | The study inclusion criteria were all household heads who were greater than or equal to 18 years and who resided in the area for at least six months. All households in the East Wollega zone were the source population for the study. The multi-stage sampling technique was applied to select the households for the study on adjusting for design effect. For qualitative, purposive sampling was carried out. Study Population |
|  |  | (*b*) *Cohort study*—For matched studies, give matching criteria and number of exposed and unexposed  *Case-control study*—For matched studies, give matching criteria and the number of controls per case |  |  |
| Variables | 7 | Clearly define all outcomes, exposures, predictors, potential confounders, and effect modifiers. Give diagnostic criteria, if applicable | 7 | Study VariablesDependent Variable Latrine utilization Independent Variables ***Socio-demography variables****:* Age, marital status, family size, educational status, presence of educated children in the household, occupation, family income, and presence of under-five children.  ***Behavioral variables:*** frequency of cleaning, hygienic condition of latrine, observable feces in the compound and latrine, knowledge about the importance of latrine use, and reasons for constructing latrine.  **Environmental Variables:** latrine service year**, s**quatting hole cover**,** presence of the door**,** frequency of latrine construction**,** the need of maintenance, number of households using well-constructed slab and superstructure latrine, implementation of CLTSH, latrine distance from the dwelling, duration of latrine owned, frequency of visit by health workers, hand washing facility and distance from health facility/kebele offices. |
| Data sources/ measurement | 8* | For each variable of interest, give sources of data and details of methods of assessment (measurement). Describe comparability of assessment methods if there is more than one group | *7-8* | **Latrine Utilization** was determined by assessing the presence of functional and improved latrines, safe disposal of children’s feces, absence of observable feces in the compound, and at least one observable sign of use (the footpath to the latrine is not covered by grass, latrine has an odor, lack of spider web in squatting hole, presence of anal cleansing material, fresh faeces in the squatting hole, or a wet slab) (37, 38).  **Functional latrines:** Latrines that were found providing services (unblocked holes/not full/ unbroken slabs, and accessible to the families) during the data collection period (39).  **Improved latrines:** Latrines such as flush/pour flush latrine connected to either sewer system, septic tanks, or pit latrine, ventilated improved pit latrine (VIPL), pit latrine with slab, and composting latrine were considered as improved latrines (40).  **Safe disposal of children’s faeces:** This variable was assessed for children less than five years and it was considered as ‘safely disposed of’ when either the child used the toilet or the family disposed of the child’s faeces in a toilet or buried it (41).  **Clean latrine**: This is the absence of fecal matter around the pit latrine and the squat hole, and properly swept latrine. It was measured based on the report of the household regarding the toilet cleaning plus observation of the feces around the squat hole and toilet. |
| Bias | 9 | Describe any efforts to address potential sources of bias | 8 | After preparing the English version of the questionnaire, it was translated into the Afaan Oromo language and then back-translated into English by another person to ensure that the originality and meaning were retained. The pre-test of the questionnaire was done on 5% of the sample in the non-selected district to identify any ambiguity, consistency, and acceptability of the questionnaire. Two days of training were given to the data collectors and supervisors. In addition, the quality of data was monitored frequently both in the field and during data entry. All completed questionnaires were examined for completeness and consistency during the interview. An incomplete and unclearly filled questionnaire was given back to the interviewer, and got complete questionnaires each day. Double data entry was done by two individuals. |
| Study size | 10 | Explain how the study size was arrived at | 6 | The study sample size was calculated by using a double population proportion formula. The proportions of latrine utilization in CLTS implemented districts (P1=54.9%) and proportion in non-CLTS districts (P2=38.7%) were considered from the study done in the Laelai Maichew district, Tigray, North Ethiopia (37). Assuming the 95% level of confidence, 80% power, 1 to 1 ratio, 10% non-response rate, and design effect = 1.5, the required sample size n was: -488 houeholds. |

Continued on next page

| Quantitative variables | 11 | Explain how quantitative variables were handled in the analyses. If applicable, describe which groupings were chosen and why | 8-9 | Quantitative data were entered into Epi Data and exported to SPSS software version 25 for data recording, cleaning, and statistical analysis. Descriptive statics using frequencies, percentages, tables, and figures were used to describe the magnitude of latrine utilization among households and other study variables. |
| --- | --- | --- | --- | --- |
| Statistical methods | 12 | (*a*) Describe all statistical methods, including those used to control for confounding | 8-9 | Bivariable logistic regression analysis was done to identify variables that were candidates for multivariable analysis. All variables that had an association on bivariable analysis at p-value <0.25 were considered for inclusion in the multivariate analysis. Then multivariable analysis was done to control the confounding effect of other variables and to identify independent factors of latrine utilization. A backward selection method was used to select the variables. Model fitness was checked using Hosmer and Lemeshow’s Test. The magnitude and direction of the relationship between the variables were expressed as odds ratios (OR) and p-value < 0.05 was used to declare the statistically significant associations. |
|  |  | (*b*) Describe any methods used to examine subgroups and interactions |  | Multi-collinearity was also checked using the correlation matrix. |
|  |  | (*c*) Explain how missing data were addressed | 9 | Missing data were excluded from the analysis. |
|  |  | (*d*) *Cohort study*—If applicable, explain how loss to follow-up was addressed  *Case-control study*—If applicable, explain how matching of cases and controls was addressed  *Cross-sectional study*—If applicable, describe analytical methods taking account of sampling strategy | 8-9 |  |
|  |  | (*e*) Describe any sensitivity analyses | 8-9 |  |
| Results | | | | |
| Participants | 13* | (a) Report numbers of individuals at each stage of study—eg numbers potentially eligible, examined for eligibility, confirmed eligible, included in the study, completing follow-up, and analysed | 9 | 461 |
|  |  | (b) Give reasons for non-participation at each stage | 5 | Households that were closed for two visits. |
|  |  | (c) Consider use of a flow diagram |  |  |
| Descriptive data | 14* | (a) Give characteristics of study participants (eg demographic, clinical, social) and information on exposures and potential confounders | 9-11 |  |
|  |  | (b) Indicate number of participants with missing data for each variable of interest | 12 | 116 are missing for frequency of cleaning. |
|  |  | (c) *Cohort study*—Summarise follow-up time (eg, average and total amount) |  |  |
| Outcome data | 15* | *Cohort study*—Report numbers of outcome events or summary measures over time |  |  |
|  |  | *Case-control study—*Report numbers in each exposure category, or summary measures of exposure |  |  |
|  |  | *Cross-sectional study—*Report numbers of outcome events or summary measures | *15-16* | *243* |
| Main results | 16 | (*a*) Give unadjusted estimates and, if applicable, confounder-adjusted estimates and their precision (eg, 95% confidence interval). Make clear which confounders were adjusted for and why they were included | 16-21 |  |
|  |  | (*b*) Report category boundaries when continuous variables were categorized |  |  |
|  |  | (*c*) If relevant, consider translating estimates of relative risk into absolute risk for a meaningful time period |  |  |

Continued on next page

| Other analyses | 17 | Report other analyses done—eg analyses of subgroups and interactions, and sensitivity analyses | 9 | Multi-collinearity was also checked using the correlation matrix with the result of no collinearity problem detected. |
| --- | --- | --- | --- | --- |
| Discussion | | | | |
| Key results | 18 | Summarise key results with reference to study objectives | 21, 25 |  |
| Limitations | 19 | Discuss limitations of the study, taking into account sources of potential bias or imprecision. Discuss both direction and magnitude of any potential bias | 24-25 | The first limitation is the cross-sectional nature of the study design which couldn’t ascertain the direction of association between the independent factors and latrine utilization. Secondly, only two districts were involved which could affect the generalizability of the findings to whole districts in the East Wollega Zone. |
| Interpretation | 20 | Give a cautious overall interpretation of results considering objectives, limitations, multiplicity of analyses, results from similar studies, and other relevant evidence | 25 | The latrine utilization in this study was found to be lower as compared to many other studies in different parts of the country and the open defecation-free mobilization. |
| Generalisability | 21 | Discuss the generalisability (external validity) of the study results | 25 |  |
| Other information | |  | | |
| Funding | 22 | Give the source of funding and the role of the funders for the present study and, if applicable, for the original study on which the present article is based | 26 | No funding obtained. |

*Give information separately for cases and controls in case-control studies and, if applicable, for exposed and unexposed groups in cohort and cross-sectional studies.

**Note:** An Explanation and Elaboration article discusses each checklist item and gives methodological background and published examples of transparent reporting. The STROBE checklist is best used in conjunction with this article (freely available on the Web sites of PLoS Medicine at http://www.plosmedicine.org/, Annals of Internal Medicine at http://www.annals.org/, and Epidemiology at http://www.epidem.com/). Information on the STROBE Initiative is available at www.strobe-statement.org.
