# Supplementary material for: Latrine utilization and associated factors among districts implementing and not-implementing community-led total sanitation and hygiene in East Wollega, Western Ethiopia: A comparative cross-sectional study
Source: PLoS One. 2023 Jul 13;18(7):e0288444. doi: 10.1371/journal.pone.0288444 (PMC10343159; doi:10.1371/journal.pone.0288444)
Supplement: Attachment — Submitted filename: PONE-D-23-00656_reviewer.pdf [file pone.0288444.s003.pdf]

PLOS ONE

Latrine Utilization and Associated Factors Among Districts Implementing and Not-Implementing Community-Led Total Sanitation and Hygiene in East Wollega, Western Ethiopia: A Comparative Cross-Sectional Study

--Manuscript Draft--

**Manuscript Number:**	PONE-D-23-00656
**Article Type:**	Research Article
**Full Title:**	Latrine Utilization and Associated Factors Among Districts Implementing and Not-Implementing Community-Led Total Sanitation and Hygiene in East Wollega, Western Ethiopia: A Comparative Cross-Sectional Study
**Short Title:**	Latrine utilization
**Corresponding Author:**	Adisu Tafari Shama<br>Wollega University<br>Nekemt, Oromia ETHIOPIA
**Keywords:**	Latrine utilization, CLTSH, Associated factors, households, East Wollega, Ethiopia
**Abstract:**	Background: Unsafe management and discharge of excreta to the environment lead to surface and groundwater contamination and human exposure to disease-causing micro-organisms. There is a paucity of the study that compares latrine utilization and associated factors among community-led total sanitation and hygiene implemented and non-implemented districts. Hence, this study aimed to determine the magnitude and associated factors of latrine utilization among households in community-led total sanitation and hygiene implemented and non-implemented Districts in East Wollega Zone, Western Ethiopia.<br>Methods: A cross-sectional study was conducted. A Multi-stage sampling technique was applied to select the 461 households. Quantitative data were collected using interviews and observations guided by a pre-structured questionnaire. Qualitative data were collected using key informant interviews. Data were entered using Epi Data and exported to SPSS software version 25 for data recording, cleaning, and statistical analysis. Bivariate logistic regression was run to identify the candidate variables at p-value <0.25. Variables that had associations with latrine utilization in the bi-variate analysis were entered together into multivariable logistic regression. An Adjusted odds ratio with a 95% confidence interval was calculated and P-value< 0.05 was used to declare a statistically significant association. Qualitative data were coded and analyzed thematically.<br>Result: The prevalence of latrine utilization in CLTSH implemented district was 53.7% [95%CI:47%, 60.3%] while it was 51.7% [95%CI:45.1%, 58.3%] in CLTSH non-implemented district. Overall, 52.7% [(95%CI:48%, 57.3%] of the households utilized their latrine. Religion [AOR=0.149(95%CI:0.044,0.506], education [AOR=3.861(95%CI:1.642,9.077], occupation, absence of children<5 years [AOR=4.724(95%CI:2.313,9.648], toilet cleaning [AOR=10.662(95%CI:5.571,20.403], frequency of latrine construction [AOR=6.441(95%CI:2.203,18.826], maintenance need [AOR=6.446(95%CI:3.023,13.744], distance from health institution [AOR=0.987(95%CI:0.978, 0.996], distance from kebele office [AOR=6.478 (95%CI:2.137,19.635], and latrine distance from dwelling [AOR=11.656(95%CI:2.108, 64.44] were the factors associated with latrine use.<br>Conclusion: The latrine utilization in this study is low as compared to other studies. Religion, education, occupation, absence of children <5 years, toilet cleaning, frequency of latrine construction, maintenance need of the toilet, distance from health institution, distance from kebele, and latrine distance from dwelling are the associated factors of latrine utilization. Both households and health workers have to work together to improve latrine utilization and the safe disposal of children's feces.
**Order of Authors:**	Adisu Tafari Shama
	Dufera Rikitu Terefa
	Edosa Tesfaye Geta
	Melese Chego Cheme

Powered by Editorial Manager® and ProduXion Manager® from Aries Systems Corporation

	Bayise Biru
	Jira Wakoya Feyisa
	Matiyos Lema
	Adisu Ewunetu
	Bikila Regassa Feyisa
	Dejene Seyoum Gebre
**Additional Information:**	
**Question**	**Response**
**Financial Disclosure**<br><br>Enter a financial disclosure statement that describes the sources of funding for the work included in this submission. Review the submission guidelines for detailed requirements. View published research articles from *PLOS ONE* for specific examples.<br><br>This statement is required for submission and **will appear in the published article** if the submission is accepted. Please make sure it is accurate.<br><br>**Unfunded studies**<br>Enter: *The author(s) received no specific funding for this work.*<br><br>**Funded studies**<br>Enter a statement with the following details:<br>• Initials of the authors who received each award<br>• Grant numbers awarded to each author<br>• The full name of each funder<br>• URL of each funder website<br>• Did the sponsors or funders play any role in the study design, data collection and analysis, decision to publish, or preparation of the manuscript?<br>• **NO** - Include this sentence at the end of your statement: *The funders had no role in study design, data collection and analysis, decision to publish, or preparation of the manuscript.*<br>• **YES** - Specify the role(s) played.<br><br>* typeset	The authors received no specific funding for this work.
**Competing Interests**<br><br>Use the instructions below to enter a	The authors have declared that no competing interests exist.

competing interest statement for this submission. On behalf of all authors, disclose any competing interests that could be perceived to bias this work—acknowledging all financial support and any other relevant financial or non-financial competing interests.<br><br>This statement is required for submission and **will appear in the published article** if the submission is accepted. Please make sure it is accurate and that any funding sources listed in your Funding Information later in the submission form are also declared in your Financial Disclosure statement.<br><br>View published research articles from *PLOS ONE* for specific examples.<br><br>**NO authors have competing interests**<br><br>Enter: *The authors have declared that no competing interests exist.*<br><br>**Authors with competing interests**<br><br>Enter competing interest details beginning with this statement:<br><br>*I have read the journal's policy and the authors of this manuscript have the following competing interests: [insert competing interests here]*<br><br>* typeset	
**Ethics Statement**<br><br>Enter an ethics statement for this submission. This statement is required if the study involved:<br><br>• Human participants<br>• Human specimens or tissue<br>• Vertebrate animals or cephalopods<br>• Vertebrate embryos or tissues<br>• Field research	This research was done according to the Helsinki declaration regarding the research ethical issues. Ethical clearance was obtained from the Wollega University, research ethics review committee before data collection with the reference number IHSRPTTAD90/2014. Institute of Health Sciences wrote the official letter to the East Wollega health department. Also, letter of cooperation was taken from the zonal health department and oral permission was obtained from the district Health offices before the start of data collection. All necessary measures were made to guard against any form of harm and discomfort to the study subjects. The study purpose, risks, and benefits were explained in the local language for the participants. Their informed, voluntary, written and signed consent, in the end, was sought and participants thumb printed consent to participate in the study. Confidentiality was also guarded by making sure that the study participants will not be represented by their names. In addition, password

Powered by Editorial Manager® and ProduXion Manager® from Aries Systems Corporation

Write "N/A" if the submission does not require an ethics statement.<br><br>General guidance is provided below. Consult the submission guidelines for detailed instructions. **Make sure that all information entered here is included in the Methods section of the manuscript.**<br><br>**Format for specific study types**<br><br>**Human Subject Research (involving human participants and/or tissue)**<br>• Give the name of the institutional review board or ethics committee that approved the study<br>• Include the approval number and/or a statement indicating approval of this research<br>• Indicate the form of consent obtained (written/oral) or the reason that consent was not obtained (e.g. the data were analyzed anonymously)<br><br>**Animal Research (involving vertebrate animals, embryos or tissues)**<br>• Provide the name of the Institutional Animal Care and Use Committee (IACUC) or other relevant ethics board that reviewed the study protocol, and indicate whether they approved this research or granted a formal waiver of ethical approval<br>• Include an approval number if one was obtained<br>• If the study involved *non-human primates*, add *additional details* about animal welfare and steps taken to ameliorate suffering<br>• If anesthesia, euthanasia, or any kind of animal sacrifice is part of the study, include briefly which substances and/or methods were applied<br><br>**Field Research**<br><br>Include the following details if this study involves the collection of plant, animal, or other materials from a natural setting:<br>• Field permit number<br>• Name of the institution or relevant body that granted permission	protection of soft data and the use of a key and lock for hard copy data was employed to guarantee confidentiality.

Powered by Editorial Manager® and ProduXion Manager® from Aries Systems Corporation

**Data Availability**<br><br>Authors are required to make all data underlying the findings described fully available, without restriction, and from the time of publication. PLOS allows rare exceptions to address legal and ethical concerns. See the PLOS Data Policy and FAQ for detailed information.<br><br>A Data Availability Statement describing where the data can be found is required at submission. Your answers to this question constitute the Data Availability Statement and **will be published in the article**, if accepted.<br><br>**Important:** Stating 'data available on request from the author' is not sufficient. If your data are only available upon request, select 'No' for the first question and explain your exceptional situation in the text box.<br><br>Do the authors confirm that all data underlying the findings described in their manuscript are fully available without restriction?	Yes - all data are fully available without restriction
**Describe where the data may be found in full sentences. If you are copying our sample text, replace any instances of XXX with the appropriate details.**<br><br>• If the data are **held or will be held in a public repository**, include URLs, accession numbers or DOIs. If this information will only be available after acceptance, indicate this by ticking the box below. For example: *All XXX files are available from the XXX database (accession number(s) XXX, XXX.*).<br>• If the data are all contained **within the manuscript and/or Supporting Information files**, enter the following: *All relevant data are within the manuscript and its Supporting Information files.*<br>• If neither of these applies but you are able to provide **details of access elsewhere**, with or without limitations, please do so. For example:	The datasets used and/or analyzed during the current study are available from the corresponding author (Adisu Tafari Shama) upon reasonable request. The email address for the contact person for data is :adisuteferi1906@gmail.com

Data cannot be shared publicly because of [XXX]. Data are available from the XXX Institutional Data Access / Ethics Committee (contact via XXX) for researchers who meet the criteria for access to confidential data.<br><br>*The data underlying the results presented in the study are available from (include the name of the third party and contact information or URL).*<br>• This text is appropriate if the data are owned by a third party and authors do not have permission to share the data.<br><br>* typeset	
Additional data availability information:	

Powered by Editorial Manager® and ProduXion Manager® from Aries Systems Corporation

Manuscript Click here to access/download;Manuscript;00_Manuscript.docx

Latrine Utilization and Associated Factors Among Districts Implementing and Not-Implementing Community-Led Total Sanitation and Hygiene in East Wollega, Western Ethiopia: A Comparative Cross-Sectional Study

Adisu Tafari Shama[1], Dufera Rikitu Terefa[1], Edosa Tesfaye Geta[1], Melese Chego Cheme[1], Bayise Biru[1,2], Jira Wakoya Feyisa[1], Matiyos Lema[1], Adisu Ewunetu[1], Bikila Regassa Feyisa[1,3], Dejene Seyoum Gebre[1]

Affiliations

[1] Department of public health, Institute of health sciences, Wollega University, Nekemt, Ethiopia

[2]Department of Human Nutrition and Dietetics, Faculty of Public Health, Jimma University, Jimma, Ethiopia

[3]Department of Epidemiology, Faculty of Public Health, Jimma University, Jimma, Ethiopia

Corresponding Author

Adisu Tafari Shama: adisuteferi1906@gmail.com

Abstract

Background: Unsafe management and discharge of excreta to the environment lead to surface and groundwater contamination and human exposure to disease-causing micro-organisms. There is a paucity of the study that compares latrine utilization and associated factors among community-led total sanitation and hygiene implemented and non-implemented districts. Hence, this study aimed to determine the magnitude and associated factors of latrine utilization among households in community-led total sanitation and hygiene implemented and non-implemented Districts in East Wollega Zone, Western Ethiopia.

Methods: A cross-sectional study was conducted. A Multi-stage sampling technique was applied to select the 461 households. Quantitative data were collected using interviews and observations guided by a pre-structured questionnaire. Qualitative data were collected using key informant interviews. Data were entered using Epi Data and exported to SPSS software version 25 for data recording, cleaning, and statistical analysis. Bivariate logistic regression was run to identify the candidate variables at p-value <0.25. Variables that had associations with latrine utilization in the bi-variate analysis were entered together into multivariable logistic regression. An Adjusted odds ratio with a 95% confidence interval was calculated and P-value< 0.05 was used to declare a statistically significant association. Qualitative data were coded and analyzed thematically.

Result: The prevalence of latrine utilization in CLTSH implemented district was 53.7% [95%CI:47%, 60.3%] while it was 51.7% [95%CI:45.1%, 58.3%] in CLTSH non-implemented district. Overall, 52.7% [(95%CI:48%, 57.3%] of the households utilized their latrine. Religion [AOR=0.149(95%CI:0.044,0.506], education [AOR=3.861(95%CI:1.642,9.077], occupation, absence of children<5 years [AOR=4.724(95%CI:2.313,9.648], toilet cleaning [AOR=10.662(95%CI:5.571,20.403], frequency of latrine construction [AOR=6.441(95%CI:2.203,18.826], maintenance need [AOR=6.446(95%CI:3.023,13.744], distance from health institution [AOR=0.987(95%CI:0.978, 0.996], distance from kebele office [AOR=6.478 (95%CI:2.137,19.635], and latrine distance from dwelling [AOR=11.656(95%CI:2.108, 64.44] were the factors associated with latrine use.

Conclusion: The latrine utilization in this study is low as compared to other studies. Religion, education, occupation, absence of children <5 years, toilet cleaning, frequency of latrine construction, maintenance need of the toilet, distance from health institution, distance from kebele, and latrine distance from dwelling are the associated factors of latrine utilization. Both households

and health workers have to work together to improve latrine utilization and the safe disposal of children's feces.

Keywords: Latrine utilization, CLTSH, Associated factors, households, East Wollega, Ethiopia

Background

Unsafe management and discharge of excreta to the environment leads to surface and groundwater contamination and human exposure to disease causing-micro-organisms (1, 2). In other ways, a latrine is a facility that is used for the safe disposal of human faeces and urine (3). It is the lowest cost option that ensures a clean and healthful living environment both at home and in the neighborhood of users (4). The use of accessible improved latrines in households and other settings is the core strategy in water, sanitation, and hygiene (WASH) interventions and also to fight neglected tropical diseases which are affecting one billion people globally (5). Further, effective utilization of latrine can prevent diarrhea and malnutrition (6).

There is confusion around the issue of how the accessibility of improved latrines is determined. Scholars argue that estimation of access to improved latrine needs to consider the chain of the faecal sludge management (FSM) system from containment to adequate treatment as well as proper utilization and user behavior not merely the type of latrine technology. Hence, there is a need to consider adequate treatment and valorization of fecal wastes as a criterion for using improved latrines in the Sustainable development goal (SDG) era (7).

To improve latrine ownership and utilization, various approaches have been implemented by government and non-governmental organizations. For instance, community-led total sanitation (CLTS), pioneered by Dr. Kamal Kar, is one of the new approaches implemented to reduce open defecation (OD) and improve hygiene and sanitation practices in different parts of developing countries. Study findings showed that CLTS was an important approach for increasing latrine ownership and utilization rates (8-12). The Ethiopian Government added the hygiene component and adapted the approach by renaming it as community-led total sanitation and hygiene (CLTSH) in 2006 to improve the sanitation and hygiene status of the community (13). The focus of CLTSH is the eradication of open defecation (OD) at a community level by generating sustained change in the collective behavior of OD practices and initiating latrine demand without outer support (14-16). The communities are enabled to observe, appraise, and analyze their OD practice and its effects. The absence of the provision of sanitation facilities is the characteristic of this CLTS (14, 17).

In the SDG, United Nations has planned to end open defecation by paying special attention to those in need (18). Globally, 2.4 billion population lack sanitary facilities, and about one billion practice open defecation (19). About 28.5% of the Ethiopian population is still practicing open defecation with the highest proportion in rural than urban areas (35.4% versus 10.1%) (20) and one out of six Ethiopian households is slipping into open defecation practices after certification for ODF (21). The studies conducted in various areas of Ethiopia revealed a 31.08%-90% prevalence of latrine utilization (22-26). This level of latrine utilization in Ethiopia is poor compared to the sustainable development goal target of ensuring 100% latrine ownership and utilization (18). Moreover, a significant proportion (56%) of rural populations is utilizing unimproved latrines which do not guarantee breaking the chain of contact with human feces and urine- the purpose of sanitation (20).

Poor latrine utilization conditions are among the major causes of public health problems in Ethiopia where children are the most vulnerable (27). Communicable diseases account for about 60% to 80% of health problems that are preventable and considerable proportions of these diseases are directly related to poor latrine utilization (28, 29). Unsanitary disposal of human excreta, together with unsafe drinking water and poor hygiene conditions contribute to 88% of diarrheal diseases. In addition, inadequate sanitation is implicated in helminthic infections, enteric fevers, and trachoma. The burden of this disease is a leading cause of morbidity and mortality, particularly in young children. Poor latrine utilization results in 5 to 12 episodes of diarrhea and between 50, 000 to 112,000 under-five children die annually in Ethiopia. Lack of access to sanitation has significant non-health consequences, especially for women and girls, including lack of security and privacy, decreased school attendance, and basic human dignity (28, 30-34). Economically, poor sanitation costs 13.5 billion Ethiopian Birr each year (35).

So far conducted studies identified that latrine utilization is affected by socio-demographic and economic, behavioral, and environmental factors (22-26).

To the authors' knowledge, even though there are studies conducted on latrine utilization in different regions of Ethiopia, there is a paucity of the study to compare the prevalence of latrine utilization among villages in CLTSH-implemented and non-implemented districts. The studies conducted did not measure specifically the utilization of safely managed latrines which is required in the SDG era. Besides, the status of CLTSH implementation and challenges are not well addressed in many of the previous studies. Therefore, this study was intended to compare the

prevalence of latrine utilization and associated factors among the CLTSH-implemented and non-implemented districts of East Wollega, Western Ethiopia.

Methods and Materials

Study Area and Period

The study was conducted in the East Wollega Zone, Oromia Regional State of Ethiopia from July 1 to 30, 2022. East Wollega is one of the 21 Zones found in the Oromia Regional State. It is located 331 km far from Addis Ababa, the capital city of Ethiopia. East Wollega zone has 17 Districts, 340 kebeles (the smallest administrative unit in the Ethiopian context), 338,022 households, and a 1,622,507 total population. Although CLTSH was implemented in all districts, it didn't reach some villages with variation from district to district. Accordingly, only one District (Gobu Sayo) has implemented the CLTSH with better reach for many of the villages while the other districts didn't reach many of their villages (36).

Study Design

A comparative community-based cross-sectional study design mixed with a qualitative method was employed.

Source Population

All households in the East Wollega zone were the source population for the study.

Study Population

All the households that have a latrine and were found in the selected districts of East Wollega were the study population.

Inclusion and Exclusion Criteria

Inclusion criteria

The study inclusion criteria were all household heads who were greater than or equal to 18 years and who resided in the area for at least six months.

Exclusion criteria

All household heads who satisfied the inclusion criteria but who were away from home and/or houses that were closed or could not be accessed at the time of the survey for two visits were excluded from the study.

Sample Size Determination

Sample Size Determination for quantitative data collection methods

The study sample size was calculated by using a double population proportion formula. The proportions of latrine utilization in CLTS implemented districts (P1=54.9%) and proportion in non-CLTS districts (P2=38.7%) were considered from the study done in the Laelai Maichew district, Tigray, North Ethiopia (37). Assuming the 95% level of confidence, 80% power, 1 to 1 ratio, 10% non-response rate, and design effect = 1.5, the required sample size n was: -

$$n = \frac{2*[\left(Zcv\sqrt{2p(1-p)}+Zpower\sqrt{P1}(1-P1)+P2\ (1-P2)\right)^2]}{(P1-P2)^2}\ +10\%\ \text{non-response rate},$$

Whereas, n=the sample size

Zcv = Z critical value for alpha which is 1.96 for this case, Zpower = Z value for 1-beta which is 0.842 in this study

P1= expected proportion of latrine utilization in sample one, P2 = expected proportion of latrine utilization for sample two, $p = \frac{P1+P2}{2}$

This yielded a total of 325 households. Then after multiplying by 1.5 for design effect, 488 households (244 households at each site) were considered to be included in the study.

For the qualitative method, six key informants involving WASH experts from the Zonal health department, experts from the districts, and health extension workers were interviewed.

Sampling Procedure/Technique

The multi-stage sampling technique was applied to select the households for the study on adjusting for design effect. First, one district that had better the implementation of CLTSH and another district that did not reach many of its villages and implemented CLTSH were randomly selected by lottery method from the Zone. Then six Kebeles three from each CLTSH implemented and non-implemented districts were selected randomly by lottery method. From each kebeles, 3 villages were selected. The calculated sample size was proportionally allocated to the selected villages. To draw a sampling frame, the total number of households in the villages was obtained from the respective village leader (*abbaa or haadha garee*). Finally, using a systematic random sampling technique (every k^{th} number of households), the final sample size included in the study was selected. For the qualitative methods, the participants were selected purposively.

Data Collection Methods

The data were collected using both quantitative and qualitative methods. The quantitative data were collected through face-to-face interviews and observations using a pre-structured standardized questionnaire adapted from the world health organization and the united nations children's fund (38). The questionnaire comprised four sections: socio-demographic and economic characteristics, behavioral factors, environmental factors, and latrine utilization-related questions that were supported by the observation. The qualitative data were collected using the semi-structured interview guide that was prepared after reviewing different literature and consulting the experts in the field. The key informant interviews were conducted to collect qualitative data and the rapporteur wrote the notes during the interview.

For quantitative data, nine first-degree holder health professionals and three Master's degree holder health workers who can speak the Afaan Oromo language were recruited as data collectors and supervisors respectively.

Study Variables

Dependent Variable

Latrine utilization

Independent Variables

Socio-demography variables: Age, marital status, family size, educational status, presence of educated children in the household, occupation, family income, and presence of under-five children.

Behavioral variables: frequency of cleaning, hygienic condition of latrine, observable feces in the compound and latrine, knowledge about the importance of latrine use, and reasons for constructing latrine.

Environmental Variables: latrine service year**, s**quatting hole cover**,** presence of the door**,** frequency of latrine construction**,** the need of maintenance, number of households using well-constructed slab and superstructure latrine, implementation of CLTSH, latrine distance from the dwelling, duration of latrine owned, frequency of visit by health workers, hand washing facility and distance from health facility/kebele offices.

Operational definitions

Latrine Utilization The use of the latrine was assessed based on self-reporting, and the observation of proxy indicators. Accordingly, the latrine is utilized when households are having a

function and improved latrines, children's faeces are safely disposed of, no observable faeces in the compound, and at least one observable sign of use (e.g., the footpath to the latrine is not covered by grass, latrine has an odor, lack of spider web in squatting hole, presence of anal cleansing material, fresh faeces in the squatting hole, or a wet slab) (39, 40). Finally, the household was categorized as utilized or not utilized based on the above definition.

Safe disposal of children's faeces: This variable was assessed for children less than five years and it was considered as 'safely disposed of' when either the child used the toilet or the family disposed of the child's faeces in a toilet or buried it (41).

Clean latrine: This is the absence of fecal matter around the pit latrine and the squat hole, and properly swept latrine. It was measured based on the report of the household regarding the toilet cleaning plus observation of the feces around the squat hole and toilet.

Data Quality Control

After preparing the English version of the questionnaire, it was translated into the Afaan Oromo language and then back-translated into English by another person to ensure that the originality and meaning were retained. The pre-test of the questionnaire was done on 5% of the sample in the non-selected district to identify any ambiguity, consistency, and acceptability of the questionnaire. Then necessary corrections were made before the actual data collection to make it ready for final data collection.

Two days of training were given to the data collectors and supervisors on the topic, how to collect the data, when and how to make an interview, and ethical issues emphasizing the importance of the safety of participants and data quality. In addition, the quality of data was monitored frequently both in the field and during data entry. This was done in the field through close supervision of interviewers. Data quality tables were utilized. All completed questionnaires were examined for completeness and consistency during the interview. An incomplete and unclearly filled questionnaire was given back to the interviewer, and got complete questionnaires each day. Double data entry using a programmed computer software package was done by two individuals.

Data Processing and Analysis

Quantitative data were entered into Epi Data and exported to SPSS software version 25 for data recording, cleaning, and statistical analysis. Descriptive statics using frequencies, percentages, tables, and figures were used to describe the magnitude of latrine utilization among households. Bivariable logistic regression analysis was done to identify variables that were candidates for

multivariable analysis. All variables that had an association on bivariable analysis at p-value <0.25 were considered for inclusion in the multivariate analysis. Then multivariable analysis was done to control the confounding effect of other variables and to identify independent factors of latrine utilization. A backward selection method was used to select the variables. Multi-collinearity was also checked using the correlation matrix with the result of no collinearity problem detected. Model fitness was checked using Hosmer and Lemeshow's Test and the result showed a p-value of 0.083 which indicated that the model fitted the data. The magnitude and direction of the relationship between the variables were expressed as odds ratios (OR) and p-value < 0.05 was used to declare the statistically significant associations. Missing data were excluded from the analysis. In another way, the qualitative data were analyzed thematically. To do this, the notes taken by the rapporteur were read repeatedly, transcribed, and themes were created.

Result

Of the 488, 461 households (229 from the villages in CLTSH-implemented versus 232 from the CLTSH non-implemented districts participated in this study with a response rate of 94.5%. The mean age with standard deviation (SD) of the respondent was 44.53 ±11.36 years. The average family size of the household was 5.34± 1.94 SD persons. The average walking time from home to health institutions was 73.64 ±42.704 SD minutes (Table 1).

Table 1: Socio-demographic and economic characteristics of the households that involved in the study of latrine utilization, 2022 (461)

Variable category		CLTSH ever implemented		Total; Frequency	(%)
		Yes (n=229); frequency (%)	No (n=232); frequency (%)		
Age in years	18-25	4 (1.7)	7 (3.0)	11	2.4
	26-30	20 (8.7)	30 (12.9)	50	10.8
	31-35	30 (13.1)	31 (13.4)	61	13.2
	36-40	42 (18.3)	39 (16.8)	81	17.6
	41-45	37 (16.2)	28 (12.1)	65	14.1
	46-50	42 (18.3)	41 (17.7)	83	18.0
	51-55	17 (7.4)	16 (6.9)	33	7.2

	56-60	22 (9.6)	19 (8.2)	41	8.9
	>60	15 (6.6)	21 (9.1)	36	7.8
Gender	Female	35 (15.3)	25 (10.8)	60	13.0
	Male	194 (84.7)	207 (89.2)	401	87.0
Religion	Protestant	75 (32.8)	76 (32.8)	151	32.8
	Orthodox	133 (58.1)	141 (60.8)	274	59.4
	Catholic	9 (3.9)	10 (4.3)	19	4.1
	Muslim	10 (4.4)	5 (2.2)	15	3.3
	Other	2 (0.9)	0 (0)	2	.4
Marital status	Single	12 (5.2)	11 (4.7)	23	5.0
	Widowed	19 (8.3)	11 (4.7)	30	6.5
	Divorced/Separated	19 (8.3)	19 (8.2)	38	8.2
	Married	179 (78.2)	191 (82.3)	370	80.3
Educational status of the respondent	Cannot read and write	72 (31.4)	68 (29.3)	140	30.4
	can read and write	52 (22.7)	69 (29.7)	121	26.2
	Primary school (1-8)	84 (36.7)	78 (33.6)	162	35.1
	High school (9-12)	14 (6.1%)	9 (3.9)	23	5.0
	college/university	7 (3.1%)	8 (3.4)	15	3.3
Educational status of the mother	Cannot read and write	90 (39.3)	96 (41.4)	186	40.3
	can read and write	59 (25.8)	78 (33.6)	137	29.7
	Primary school (1-8)	66 (28.8)	50 (21.6)	116	25.2
	High school (9-12)	9 (3.9)	5 (2.2)	14	3.0
	college/university	5 (2.2)	3 (1.3)	8	1.7
Have school children/educated children	No	65 (28.4)	60 (25.9)	125	27.1
	Yes	164 (71.6)	172 (74.1)	336	72.9
Occupation of household head	Farmer	202 (88.2)	207 (89.2)	409	88.7
	daily laborer	12 (5.2)	11 (4.7%)	23	5.0
	Merchant	7 (3.1)	5 (2.2%)	12	2.6
	gov employee	5 (2.2)	6 (2.6)	11	2.4
	NGO employee	3 (1.3)	3 (1.3)	6	1.3
Occupation of the mother	Farmer	119 (52.0)	120 (51.7)	239	51.8
	Housewife	93 (40.6)	93 (40.1)	186	40.3
	daily laborer	7 (3.1)	8 (3.4)	15	3.3

	Merchant	2 (0.9)	0 (0)	2	.4
	gov employee	5 (2.2)	8 (3.4)	13	2.8
	NGO employee	2 (0.9)	2 (0.9)	4	.9
	Others	1 (0.4)	1 (0.4)	2	.4
Income	<=1000	95 (41.5)	83 (35.8)	178	38.6
	1001-2000	50 (21.8)	46 (19.8)	96	20.8
	2001-3000	36 (15.7)	44 (19)	80	17.4
	3001-4000	31 (13.5)	32 (13.8)	63	13.7
	4001-5000	13 (5.7)	19 (8.2)	32	6.9
	>5000	4 (1.7)	8 (3.4)	12	2.6
Have children <5 years	No	111 (48.5)	106 (45.7)	217	47.1
	Yes	118 (51.5)	126 (54.3)	244	52.9
Family size	≤5	131 (57.2)	139 (59.9)	270	58.6
	>5	98 (42.8)	93 (40.1)	191	41.4

About half of the households-49.3% in CLTSH implemented villages and 50.4%% in CLTSH non-implemented villages clean their toilet. The majority of the households 149 (65.1%) in CLTSH implemented villages and 143 (61.6%) in CLTSH non-implemented villages were motivated to construct the toilet by primary health workers. Feces were observed in about one-third-34.9% of compounds of the study households while majority-427 (92.6%) of the households know the disease preventability of latrine utilization (Table 2).

Table 2: Behavioral factors of latrine utilization in East Wollega, Western Ethiopia, 2022 (n=461)

Variable category		CLTSH ever implemented		Total; Freq.	(%)
		Yes (n=229); frequency (%)	No (n=232); frequency (%)		
Clean toilet	Not clean	116 (50.7)	115 (49.6)	231	50.1
	Clean	113 (49.3)	117 (50.4)	230	49.9
Frequency of cleaning	Daily	11 (4.8)	13 (5.6)	24	5.2
	Sometimes	100 (43.7)	117 (50.4)	217	47.1
	Rarely	71 (31.0)	57 (24.6)	128	27.8

	Do not clean at all	47 (20.5)	45 (19.4)	92	20.0
Motivation/reason to construct latrine	Self-initiation	28 (12.2)	30 (12.9)	58	12.6
	Kebele leaders	50 (21.8)	59 (25.4)	109	23.6
	Primary health workers	149 (65.1)	143 (61.6)	292	63.3
	Peers	2 (0.9)	0 (0)	2	0.4
Observable feces in the compound	No	152 (66.4)	148 (63.8)	300	65.1
	Yes	77 (33.6)	84 (36.2)	161	34.9
Knowledge about disease preventability of latrine usage	No	20 (8.7)	14 (6.0)	34	7.4
	Yes	209 (91.3)	218 (94.0)	427	92.6
Know preventability by avoiding the probability of feces contact with fingers		115 (55)	133 (61)	248	58
Know preventability by avoiding contact of feces with fluids		108 (51.6)	109 (50)	217	50.8
Know preventability by avoiding contact of feces with fomites/materials		43 (205)	29 (13.3)	72	16.86
Know preventability by avoiding contact of feces with flies		36 (17.22)	27 (12.3)	63	14.75
Know preventability by avoiding contact of feces with food		79 (37.79)	87 (39.9)	166	38.87

More than three quarters-365 (79.2%) of the households have hand washing facilities near their toilet. About 379 (82.2%) of the households in the study reported that they wash their hands after the toilet visits. The average water consumption for hand washing in a day was 6.67 ±11.35 SD liters among the households (Figure 1).

Figure 1: hand washing facility and hand washing practice of the study households, 2022 (n=461)

Of the 365 households that have hand washing facilities, water is observed in the hand washing facilities of 343 (93.97%) households. Similarly, detergents are observed for 207 (56.7%) households having hand-washing facilities (Figure 2).

Figure 2: Availability of water and detergents for the hand washing among the households that participated in the study of latrine utilization, 2022 (n=365).

More than half-54.1% of toilets in CLTSH-implemented villages and about two-third-66.8% of toilets in CLTSH no-implemented villages have no door. Majority of the toilets; 82.5% in CLTSH implemented villages and 83.2% in CLTSH non-implemented villages have squat holes covered by earth. About 134 (58.5%) households in CLTSH-implemented villages and 134 (57.8%) in CLTSH non-implemented villages constructed toilets for the second time. Regarding the maintenance need of a toilet, about two-thirds of the toilets;147 (64.2%) in CLTSH implemented villages, 160 (69.0%) in CLTSH non-implemented villages and 307 (66.6%) of the total sample households reported that their toilets need maintenance. Nearly two-thirds-302 (65.5%) of the households reported that they are located at a medium distance from the kebele office (Table 3).

Table 3: Environmental factors of latrine utilization in East Wollega Zone, western Ethiopia, 2022 (n=461).

Variable category		CLTSH ever implemented		Total; Frequency	(%)
		Yes (n=229); frequency (%)	No (n=232); frequency (%)		
Toilet has door	No	124(54.1)	155 (66.8)	279	60.5
	Yes	105 (45.9)	77 (33.2)	182	39.5
Squat hole covered	Yes, by slab	20 (8.7)	16 (6.9)	36	7.8
	Yes, by earth	189 (82.5)	193 (83.2)	382	82.9

	Open	20 (8.7)	23 (9.9)	43	9.3
Frequency of latrine construction	One	54 (23.6)	64 (27.6)	118	25.6
	Two	134 (58.5)	134 (57.8)	268	58.1
	Three	36 (15.7)	31 (13.4)	67	14.5
	4 and above	5 (2.2)	3 (1.3)	8	1.7
Source of information regarding latrine construction	Health professionals	187 (81.7)	200 (86.2)	387	83.9
	Through sanitation campaign	5 (2.2)	5 (2.2)	10	2.2
	Family members	9 (3.9)	8 (3.4)	17	3.7
	Mass media	8 (3.5)	8 (3.4)	16	3.5
	Neighborhoods	11 (4.8)	2 (0.9)	13	2.8
	Students	9 (3.9)	9 (3.9)	18	3.9
Toilet needs maintenance	No	82 (35.8)	72 (31.0)	154	33.4
	Yes	147 (64.2)	160 (69.0)	307	66.6
Part of toilet that needs maintenance (n=307)	Super structure	16 (	12 ()	28	9.1
	Slab	41	60	101	32.9
	Roof	18	26	44	14.3
	Mixed	69	60	129	42
	Other	3	2	5	1.6
Distance from kebele office	Near	25 (10.9)	29 (12.5)	54	11.7
	Medium	157 (68.6)	145 (62.5)	302	65.5
	Too far	47 (20.5)	58 (25.0)	105	22.8
Latrine Distance from dwelling in meter	>10	16 (7.0)	8 (3.4)	24	5.2
	≤10	213 (93.0)	224 (96.6)	437	94.8

Latrine Distance from water source in meter	≤15	12 (5.2)	9 (3.9)	21	4.6
	>15	217 (94.8)	223 (96.1)	440	95.4
Duration since toilet owned	≤2 years	111 (48.5)	123 (53.0)	234	50.8
	>2years	118 (51.5)	109 (47.0)	227	49.2
Distance from health institution in minutes	Near (≤60)	126 (55.0)	140 (60.3)	266	57.7
	Medium (61-120)	92 (40.2)	80 (34.5)	172	37.3
	Far (>120)	11 (4.8)	12 (5.2)	23	5.0
Frequency of supervision by health professionals in a month	No supervision	12 (5.2)	7 (3.0)	19	4.1
	One	53 (23.1)	45 (19.4)	98	21.3
	Two	149 (65.1)	159 (68.5)	308	66.8
	Three and above	15 (6.6)	21 (9.1)	36	7.8
Frequency of supervision by local leaders in a week	No supervision	22 (9.6)	22 (9.5)	44	9.5
	One	155 (67.7)	142 (61.2)	297	64.4
	Two	52 (22.7)	68 (29.3)	120	26.0
Hand washing facility	Yes	179 (78.2)	186 (80.2)	365	79.2
	No	50 (21.8)	46 (19.8)	96	20.8

229 (49.67) households in this study were from the CLTSH-implemented villages.

From the qualitative data, good progress in CLTSH implementation during the initial time was identified. In this regard, the 35 years old male water, sanitation, and hygiene focal person said that “CLTSH was reached almost all kebeles. However, there might be villages that are not reached. During the first time, it was progressing well. But nowadays, it is not working as it is impossible to hide. For the success of the session, you have to make them feel shame, fear, and disgust. When the residents of the villages in which the approach is planned to be implemented

already get the information from their neighbor villages in which the CLTSH is implemented, you cannot create those feelings."

A 34 male who was the coordinator of communicable diseases control-related activities at the District level addressed that "the implementation of CLTSH during the initial time was good. Because it was possible to hide what was going to be done to accomplish the trigger target". However, as time goes on, the information disseminates rapidly and it became difficult to follow the procedures."

A 34 male key informant added on this issue that "currently, the ODF replaced the CLTSH. The concept is almost similar to that of CLTSH but it doesn't fully follow the CLTSH procedures. Although the ODF is progressing well, still it has challenges. Because it is under political pressure and there is an occasion when the professionals are urged to verify as open defecation free without fulfilling the criteria. Politicians say that health professionals have the problem. Why don't you verify? Our Woreda is lagging while X or Y woreda is accomplishing good?"

Information contamination, phasing out of non-governmental organizations (NGOs), and lack of post-ignition monitoring and follow-up were the challenges related to CLTSH implementation

The health extension worker forwarded this idea. "It is impossible to hide the CLTSH procedures because the neighboring villages can get the information once the CLTSH is implemented in another village. Our community is interlinked with different social activities." One male key informant responded on this issue as "in addition to information contamination, the CLTSH was NGO driven initially. When that NGO phased out, the approach failed to function". It is when the facilitators of CLTSH do not return to the villages for monitoring and follow-up after the ignition phase of the CLTSH. In this regard, the 34 years old personnel from the district health official said that "we had the problem of returning to the community to monitor the status of their performance.

Once we facilitated the implementation of CLTSH in that community, we do not go for follow-up due to different reasons even when the community invite us. This in turn affected the success of the CLTSH."

The prevalence of latrine utilization in CLTSH implemented district was 123 (53.7% [95%CI:47%, 60.3%]) while it was 120 (51.7% [95%CI:45.1%, 58.3%]) in CLTSH non-implemented district (Figure 3).

Figure 3: Comparison of latrine utilization in CLTSH implemented and non-implemented districts of East Wollega Zone, Western Ethiopia, 2022

Overall, more than have-243 (52.7% [(95%CI:48%, 57.3%]) of the households utilized their latrine (figure 4).

Figure 4: utilization of latrine among the households that participated in the study of latrine utilization and associated factors in East Wollega, Western Ethiopia, 2022 (n=461)

In bivariable logistic regression, 12 variables showed a significant association with latrine utilization in CLTSH non-implemented districts. These variables are age 36-40 as compared to 18-25 years, family size, income, having of children <5 years, occupation of household head, cleaning toilet, motivation/reason to construct latrine, frequency of latrine construction, maintenance need, distance from health institution in minutes, distance from kebele office and hand washing facility.

Nonetheless, only five factors such as having children <5 years**,** cleaning of toilet, frequency of latrine construction, maintenance need of the toilet, and distance from kebele office maintained a significant association in multivariable logistic regression (Table 4).

Table 4: Factors associated with latrine utilization among CLTSH non-implemented district in East Wollega, Western Ethiopia, 2022 (n=232)

Variables	Category	Latrine utilized					
		No=N (%)	Yes=N (%)	P-value	COR with 95%CI	P-value	AOR with 95%CI

Have children <5 years	No	27 (24.1%)	79 (65.8%)	0.000*	6.066 (3.416, 10.772)	0.001**	5.194 (1.880, 14.354)
	Yes	85 (75.9%)	41 (34.2%)	Ref	Ref	Ref	Ref
Clean toilet	Not clean	88(78.6%)	27(22.5%)		Ref		
	Clean	24(21.4%)	93(77.5%%)	0.000*	12.630 (6.778-23.532)	0.000**	15.102 (5.893, 38.699)
Frequency of latrine construction	One	29 (25.9%)	35 (29.2%)	Ref	Ref	Ref	Ref
	Two	73 (65.2%)	61 (50.8%)	.228	0.692 (0.381, 1.259)	0.333	1.648 (0.600, 4.528)
	Three	9 (8.0%)	22 (18.3%)	.132*	2.025 (0.808, 5.075)	0.019*	5.754 (1.327, 24.937)
	4 and above	1 (0.9%)	2 (1.7%)	.686	1.657 (0.143, 19.211)	0.379	0.161 (0.003, 9.375)
Maintenance need	No	20 (17.9%)	52 (43.3%)	0.000*	3.518 (1.924, 6.432)	0.006**	4.221 (1.498, 11.890)
	Yes	92 (82.1%)	68 (56.7%)	Ref	Ref	Ref	Ref
Distance from kebele office	Near	12 (10.7%)	17 (14.2%)	.023*	2.908 (1.159, 7.298)	0.001**	13.805 (2.818, 67.645)
	Medium	61 (54.5%)	84 (70.0%)	.001*	2.827 (1.491, 5.360)	0.001**	7.188 (2.190, 23.594)
	Too far	39 (34.8%)	19 (15.8%)	Ref	Ref	Ref	Ref

COR=Crude odds ratio, AOR=adjusted odds ratio, CI=confidence interval, * = p-value<0.25, ** p-value<0.05, Ref = Reference, N= Number

For the CLTSH implemented district, 17 variables such as religion, age 36-40 as compared to 18-25 years, family size, educational status of the respondent, educational status of the mother, having of educated/school children, income, having of children <5 years, occupation of the mother, cleaning toilet, motivation/reason to construct latrine, frequency of latrine construction, maintenance need, distance from health institution in minutes, distance from kebele office, latrine distance from dwelling in meter and hand washing facility associated with latrine utilization in bivariable logistic regression.

Nonetheless, only 9 factors like religion, educational status of the mother, having of children <5 years, cleaning of toilet, frequency of latrine construction, maintenance need, motivation/reason to construct latrine, latrine distance from dwelling and distance from kebele office maintained the significant association in multivariable logistic regression (Table 5).

Table 5: Factors associated with latrine utilization among CLTSH-implemented district in East Wollega, Western Ethiopia, 2022 (n=229)

Variables	Category	Latrine utilized					
		No=N (%)	Yes=N (%)	P-value	COR with 95%CI	P-value	AOR with 95%CI
Religion	Protestant	33 (31.1%)	42 (34.1%)	Ref	Ref	Ref	Ref
	orthodox	58 (54.7%)	75 (61.0%)	.956	1.016 (0.574,1.797)	0.116	.286 (0.060, 1.364)
	Others (Catholic, Muslim and Adventist)	15 (14.2%)	6 (4.9%)	.031*	0.314 (0.110, 0.899)	0.008**	0.053 (0.006, 0.463)
Educational status of the mother	Cannot read and write	33 (31.1%)	57 (46.3%)	Ref	Ref	Ref	Ref

	can read and write	29 (27.4%)	30 (24.4%)	0.132	0.599	(0.308-1.166)	0.867	0.883 (0.205, 3.799)
	Primary school (1-8)	36 (34.0%)	30 (24.4%)	.027	0.482	(0.253-0.921)	0.004**	0.095 (0.019, 0.479)
	High school and above	8 (7.5%)	6 (4.9%)	.152	0.434	(0.139-1.360)	0.435	0.148 (0.001, 17.950)
Have children <5 years	No	33 (31.1%)	78 (63.4%)	0.000*	3.834	(2.210, 6.653)	0.013**	5.030 (1.399, 18.081)
	Yes	73 (68.9%)	45 (36.6%)	Ref	Ref		Ref	Ref
Clean toilet	Not clean	86 (81.1%)	30(24.4%)	Ref	Ref		Ref	Ref
	clean	20(18.9%)	93 (75.6%)	0.000*	13.330	(7.04-25.211)	0.000**	15.152 (3.884, 59.105)
Motivation/reason to construct latrine	Self-initiation	5 (4.7%)	23 (18.7%)	.002*	4.919	(1.776,13.630)	0.004**	0.030 (0.003, 0.324)
	Kebele leaders and peers	24 (22.6%)	28 (22.8%)	.493	1.248	(0.663, 2.349)	0.282	0.414 (0.083, 2.064)
	Primary health workers	77 (72.6%)	72 (58.5%)	Ref	Ref		ref	ref
Frequency of latrine construction	One	23 (21.7%)	31 (25.2%)	Ref	Ref		Ref	Ref
	Two	74 (69.8%)	60 (48.8%)	.118	0.602	(0.318, 1.138)	0.057	3.789 (0.962, 14.926)
	Three	8 (7.5%)	28 (22.8%)	.050*	2.597	(1.001, 6.735)	0.003**	27.698 (3.131,

							244.995)
	4 and above	1 (0.9%)	4 (3.3%)	.345	2.968 (0.311 28.347)	0.461	3.948 (0.103, 151.681)
Maintenance need	No	17 (16.0%)	65 (52.8%)	0.000 *	5.867 (3.131,10.995)	0.000 **	38.391 (6.016, 245.002)
	Yes	89 (84.0%)	58 (47.2%)	Ref	Ref	Ref	Ref
Distance from kebele office	Near	7 (6.6%)	18 (14.6%)	0.030 *	3.184 (1.119, 9.056)	0.001 **	48.178 (4.761, 487.515)
	Medium	73 (68.9%)	84 (68.3%)	0.290	1.425 (0.740, 2.743)	0.192	2.742 (0.603, 12.464)
	Too far	26 (24.5%)	21 (17.1%)	Ref	Ref	Ref	Ref
Latrine Distance from dwelling in meter	>10	13 (12.3%)	3 (2.4%)	Ref	Ref	Ref	Ref
	≤10	93 (87.7%)	120 (97.6%)	0.009 *	5.591 (1.548, 20.196)	0.018 **	30.751 (1.786, 529.593)

COR=Crude odds ratio, AOR=adjusted odds ratio, CI=confidence interval, * = p-value<0.25, ** p-value<0.05, Ref = Reference, N= Number

Eighteen (18) variables showed an association with latrine utilization in bivariable logistic regression during the overall analysis of the sampled households. Age of the household head, religion, family size, educational status of the respondent, educational status of the mother, having school children/educated children, absence of children <5 years, occupation of household head,

occupation of the mother, toilet cleaning, motivation/reason to construct latrine, knowledge about disease preventability of latrine usage, frequency of latrine construction, maintenance need of toilet, distance from health institution in minutes, distance from kebele office, latrine distance from dwelling in meter and hand washing facility.

However, only ten (10) variables showed significant association in the multivariable logistic regression. religion, educational status of the respondent, occupation of the mother, absence of children <5 years, toilet cleaning, frequency of latrine construction, current maintenance need of the toilet, distance from health institution in minutes, distance from kebele office, and latrine distance from the dwelling.

Those who were in other religious categories (catholic, Muslim, and Adventist) were 85.1% (AOR=0.149;95%CI:0.044, 0.506) less likely to utilize latrine than protestant followers. The odds of latrine utilization was 3.86 (AOR=3.861;95%CI:1.642, 9.077) times more likely for the households whose heads can write and read as compared to those who are unable to read and write. Mothers who were housewives in occupation were two times (AOR=2.440;95%CI:1.028, 5.792) more likely to utilize latrines than those who were farmers.

Households in which there are no under-five children utilize latrines 4.7 (AOR=4.724;95%CI:2.313, 9.648) times more likely than their counterparts. The odds of latrine utilization was 10.6 times more likely (AOR=10.662;95%CI:5.571, 20.403) for those households who clean their toilet when compared with their counterparts. Households that constructed toilets three times utilized latrines 6 times more likely (AOR=6.441;95%CI:2.203,18.826) as compared to the first-time toilet construction. Similarly, the odds of household latrine utilization is 6 times higher (AOR=6.446;95%CI:3.023,13.744) for the latrine that doesn't need maintenance currently when compared to its counterpart. In another way, when the distance of the household from the health institutions increases by one minute, the odds of latrine utilization decrease by 1.3% (AOR=0.987;95%CI:0.978, 0.996). Those households that were near to kebele office had 6.5 times more odds of latrine utilization (AOR= 6.478;95%CI:2.137,19.635) as compared to those too far from the kebele office. Besides, households located at a medium distance from the kebele office had 4 times more odds of latrine utilization (AOR =4.163;95%CI:1.818, 9.531). Households whose latrine is ≤10 meters from the dwelling had 2.1 times increased odds of latrine utilization (AOR=11.656;95%CI:2.108, 64.440) as compared to their counterparts (Table 6).

Table 6: Factors associated with latrine utilization among CLTSH-implemented and non-implemented districts in East Wollega, Oromia, Ethiopia, 2022 (n=461).

Variables	Category	Latrine utilized					
		No=N (%)	Yes=N (%)	P-value	COR with 95%CI	P-value	AOR with 95%CI
Age category of the respondent	18-25	4 (1.8%)	7 (2.9%)	Ref	Ref		
	26-30	28(12.8%)	22 (9.1%)	0.245	0.449 (0.116-1.731)		
	31-35	31 (14.2%)	30 (12.3%)	0.382	0. 553(0.147-2.085)		
	36-40	58 (26.6%)	23 (9.5%)	0.028*	0.227 (0.061-0.848)		
	41-45	27 (12.4%)	38 (15.6%)	0.747	0.804 (0.214-3.022)		
	46-50	32 (14.7%)	51 (21.0%)	0.888	0.911(0.247-3.360)		
	51-55	17 (7.8%)	16 (6.6%)	0.387	0.538 (0.132-2.193)		
	56-60	12 (5.5%)	29 (11.9%)	0.652	1.381 (0.340-5.603)		
	>60	9 (4.1%)	27 (11.1%)	0.464	1.714(0.406-7.247)		
Gender of the household head	Female	32 (14.7%)	28 (11.5%)	0.316	0.757(.439-1.304)		
	Male	186 (85.3%)	215 (88.5%)	Ref	Ref		
Religion	Protestant	73 (33.5%)	78 (32.1%)	Ref	Ref	Ref	Ref
	Orthodox	120 (55.0%)	154 (63.4%)	.368	1.201(0.806,1.789)	0.508	0.786 (0.385, 1.604)

	Others (Catholic, Muslim and Adventist)	25 (11.5%)	11 (4.5%)	.025	0.412 (0.189,0.896)	0.002 **	0.149	(0.044, 0.506)
Marital status	Single	7 (3.2%)	16 (6.6%)	0.097	2.165 (0.871-5.386)			
	Widowed	9 (4.1%)	21 (8.6%)	0.054	2.211 (0.986-4.954)			
	Divorced/ separated	22 (10.1%)	16 (6.6%)	0.280	0.689 (0.351-1.354)			
	Married	180 (82.6%)	190 (78.2%)	Ref	Ref			
Family size	≤5	106 (48.6%)	164 (67.5%)	0.000*	2.193 (1.504-3.200)			
	>5	112 (51.4%)	79 (32.5%)	Ref	Ref			
Educational status of the respondent	Cannot read and write	59 (27.1%)	81 (33.3%)	Ref	Ref	Ref	Ref	
	can read and write	47 (21.6%)	74 (30.5%)	.588	1.147 (0.698-1.884)	0.002 **	3.861	(1.642, 9.077)
	Primary school (1-8)	98 (45.0%)	64 (26.3%)	.002 *	.476 (0.300-0.754)	0.930	0.965	(0.433, 2.151)
	High school (9-12)	11 (5.0%)	12 (4.9%)	.610	0.795 (0.328-1.924)	0.142	3.111	(0.683, 14.165)
	college/u niversity	3 (1.4%)	12 (4.9%)	.109	2.914 (0.787-10.786)	0.073	5.163	(0.858, 31.080)

Educational status of the mother	Cannot read and write	75 (34.4%)	111 (45.7%)	Ref	Ref			
	can read and write	67 (30.7%)	70 (28.8%)	.125	0.706 (0.452-1.102)			
	Primary school (1-8)	64 (29.4%)	52 (21.4%)	.012*	0.549 (0.344 0.877)			
	High school and above	12 (5.5%)	10 (4.1%)	.205	0.563 (0.231-1.370)			
Have school children/educated children	No	44 (20.2%)	81 (33.3%)	Ref	Ref			
	Yes	174 (79.8%)	162 (66.7%)	0.002*	0.506 (0.331,0.773)			
Have children <5 years	No	60 (27.5%)	157 (64.6%)	0.000*	4.807 (3.233, 7.149)	0.000 **	4.724	(2.313, 9.648)
	Yes	158 (72.5%)	86 (35.4%)	Ref	Ref	Ref	Ref	
Occupation of household head	Farmer	206 (94.5%)	203 (83.5%)	Ref	Ref			
	daily laborer	9 (4.1%)	14 (5.8%)	.298	1.579 (0.668, 3.729)			
	Others (Merchant, gov employee, NGO employee)	3 (1.4%)	26 (10.7%)	.000*	8.795 (2.621, 29.514)			
	Farmer	157 (72.0%)	82 (33.7%)	Ref	Ref	Ref	Ref	

Occupation of the mother	Housewife	51 (23.4%)	135 (55.6%)	.000*	5.068 (3.335, 7.702)	0.043 **	2.440 (1.028, 5.792)
	daily laborer	7 (3.2%)	8 (3.3%)	.143	2.188 (0.767, 6.246)	0.756	0.592 (0.022, 16.104)
	Others (Merchant, government and NGO employee)	3 (1.4%)	18 (7.4%)	.000 *	11.488 (3.288, 40.140)	0.510	0.363 (0.018, 7.404)
Clean toilet	Not clean	174 (79.8%)	57 (23.5%)	0.005 *	Ref	Ref	Ref
	clean	44 (20.2%)	186 (76.5%)	0.000*	12.904 (8.275-20.124)	0.000 **	10.662 (5.571, 20.403)
Motivation/reason to construct latrine	Self-initiation	14 (6.4%)	44 (18.1%)	.000	3.320 (1.744,6.320)		
	Kebele leaders and peers	54 (24.8%)	57 (23.5%)	.626	1.115 (0.720,1.727)		
	Primary health workers	150 (68.8%)	142 (58.4%)	Ref	Ref		
Knowledge about disease preventability of latrine usage	No	22 (10.1%)	12 (4.9%)	Ref	Ref		
	Yes	196 (89.9%)	231 (95.1%)	0.038*	2.161 (1.043,4.478)		

Door	No	126 (57.8%)	153 (63%)	Ref	Ref			
	Yes	92 (42.2%)	90 (37.0%)	0.258	0.806 (0.554, 1.171)			
Squat covered	Yes, by slab	15 (6.9%)	21 (8.6%)	.098	2.141 (0.869, 5.274)			
	Yes, by earth	177 (81.2%)	205 (84.4%)	.082	1.771 (0.931, 3.371)			
	open	26 (11.9%)	17 (7.0%)	Ref	Ref			
Frequency of latrine construction	One	52 (23.9%)	66 (27.2%)	Ref	Ref	ref	Ref	
	Two	147 (67.4%)	121 (49.8%)	.051	0.649 (0.419,1.003)	0.201	1.608	(0.777, 3.328)
	Three	17 (7.8%)	50 (20.6%)	.012*	2.317 (1.198,4.481)	0.001**	6.441	(2.203, 18.826)
	4 and above	2 (0.9%)	6 (2.5%)	.304	2.364 (0.458,12.198)	0.417	2.464	(0.279, 21.733)
Maintenance need	No	37 (17.0%)	117 (48.1%)	0.000*	4.542 (2.943,7.011)	0.000**	6.446	(3.023, 13.744)
	Yes	181 (83.0%)	126 (51.9%)	Ref	ref	ref	Ref	
Distance from health institution in minutes				0.000*	.968 (0.962, 0.975)	0.004**	0.987	(0.978, 0.996)
Distance from kebele office	Near	19 (8.7%)	35 (14.4%)	.002*	2.993 (1.511,5.929)	0.001**	6.478	(2.137, 19.635)
	Medium	134 (61.5%)	168 (69.1%)	.002*	2.037 (1.293,3.210)	0.001**	4.163	(1.818, 9.531)

	Too far	65 (29.8%)	40 (16.5%)	Ref	Ref	Ref	Ref
Latrine Distance from dwelling in meter	>10	20 (9.2%)	4 (1.6%)	Ref	Ref	Ref	Ref
	≤10	198 (90.8%)	239 (98.4%)	0.001*	6.035 (2.029,17.949)	0.005 **	11.656 (2.108, 64.440)
Latrine Distance from water source in meter	≤15	10 (4.6%)	11 (4.5%)	Ref	Ref		
	>15	208 (95.4%)	232 (95.5%)	0.975	1.014 (0.422, 2.436)		
Duration since toilet owned	≤2 years	119 (54.6%)	115 (47.3%)	Ref	Ref		
	>2years	99 (45.4%)	128 (52.7%)	0.120	1.338 (0.927,1.931)		
Frequency of supervision by health professionals in a month	No supervision	6 (2.8%)	13 (5.3%)	Ref	Ref		
	One	41 (18.8%)	57 (23.5%)	0.406	0.642 (0.225, 1.829)		
	Two	164 (75.2%)	144 (59.3%)	0.075	0.405 (0.150, 1.094)		
	Three and above	7 (3.2%)	29 (11.9%)	0.318	1.912 (0.536,6.820)		
Frequency of supervision	No supervision	17 (7.8)	27 (11.1)	Ref	Ref		

by local leaders in a week	≥1 time	201 (92.2)	216 (88.9)	0.229	0.677 (0.358,1.279)
Hand washing facility	Yes	161 (73.9%)	204 (84.0%)	0.008*	1.852 (1.173,2.924)
	No	57 (26.1%)	39 (16.0%)	Ref	Ref
CLTSH	Implemented	106 (48.6%)	123 (50.6%)	0.669	1.083 (0.751, 1.561)
	Not implemented	112 (51.4%)	123 (49.4%)	Ref	Ref

* p-value <0.25, ** p-value <0.05, COR-Crude odds ratio, AOR-Adjusted odds ratio, CI-confidence interval.

Discussion

The prevalence and associated factors of latrine utilization among households in the East Wollega Zone were assessed in this study. Accordingly, the prevalence of latrine utilization was found to be 52.7% (95%CI:48%, 57.3%). It was 51.7% in CLTSH non-implemented villages while 53.7% in CLTSH implemented villages. The value is almost similar to the national prevalence-50.02% (22). It is also consistent with the study conducted in the Laelai Maichew district of Tigray in which the latrine utilization of 54.9% among CLTSH implemented and overall latrine utilization of 46.8%-58.9% was observed (37, 42). However, the latrine utilization among CLTSH non-implemented villages in Laelai Maichew district, Tigray- 38.7% (37) was found to be lower than our finding. The current finding is also higher than the result of latrine utilization from the study in South East Zone of Tigray-37.6% (43), and the study in Chencha District, Southern Ethiopia-31.08% (26). The discrepancy could result from the difference in socio-economic status, and difference in study settings, and periods.

The overall latrine utilization prevalence in our study is lower than the reports of latrine utilization in Sebeta Hawas-68% (23), Mehal Meda town-90% (24), semi-urban areas of North Eastern Ethiopia-71.8% (39), Denbia district, Northwest Ethiopia-86.8% (25), Hulet Ejju Enessie Woreda-60.7% (41), Wondo Genet district, South Ethiopia-83.1% (44), and Hawassa town (40). The latrine

utilization among CLTSH-implemented villages in this study was also lower than the one in Gurage Zone's Community-Led Total Sanitation and Hygiene implemented kebeles-65.8% (45). The observed difference might be due to the difference in the way of latrine utilization measurement, and study settings as some of the above studies were conducted in urban and semi-urban areas. In this regard, those households that had open pits without any form of the slab were categorized as not utilizing the toilet in our study.

Those households who were followers of the protestant religion were found to utilize their toilet more likely than those in other religious categories (catholic, Muslim, and Adventist). As to the authors' knowledge, a similar finding was not observed in the literature reviewed. However, the possible explanation is that majority of the study households are protestant followers. Followers of the catholic, Muslim, and Adventist religions were few among those studied households.

Household heads who were able to write and read used their latrine about 3.86 times more likely than their counterparts. Similar results were reported from the studies conducted in SebetaHawas Woreda (23), Laelai Maichew Woreda (42), and Gurage zone (45). This is explained by the fact that education can increase the knowledge about the diseases related to improper disposal of human wastes and lead to changes in latrine utilization behavior.

Occupation of the mother was another factor that was significantly associated with latrine utilization in this study. Households with housewives were about two times more likely to utilize latrines than those whose mothers were farmers in occupation. This finding agrees with the findings of the studies conducted in Denbia (25) and Laelai Maichew (42) districts. The observed association between latrine utilization and the mother's occupation might be attributed to the large proportion of housewife participants in this study. Because the majority of the mothers in the study households were housewives. In another way, housewives might have time to keep the latrine clean and make it ready for utilization. They might easily pick and dispose of the children's feces into the toilet since they are around the home.

Absence of the under-five children in the household is one of the factors that increased the proper utilization of latrines compared to those having children under five years of age. This in line with the study conducted in Denbia district of North West Ethiopia in which the extent of latrine utilization was 62.1% less likely for households having ≤ 5 years of children (25). The possible reason is that children under five years could not use the latrine by themselves and they might

defecate in the compound which could, in turn, lead to the detection of stool in the compound during observation of the latrine utilization.

In this study, households that clean their toilet and have clean toilets had a high probability of utilizing them than their counterparts. This is relevant to the studies conducted in Denbia district (25) and Gurage (45). Although it is difficult to ascertain the direction of the association, households that care for and clean their toilet might utilize it. It might be attributed to the fact that using the unhygienically handled toilets is disgusting.

The odds of latrine utilization was about 6 times more likely for those households that constructed a toilet for the third time as compared with the first-ever toilet construction. A related finding was reported from the study conducted in Denbia district (25). The possible explanation for the observed association is that frequent latrine construction by itself might arise from the knowledge of latrine benefits and continuous use. Frequent latrine construction might be an indication of persistent latrine use.

The odds of household latrine utilization is 6 times higher for the latrine that doesn't need maintenance currently when compared to its counterpart. It might be difficult to utilize the latrine which needs maintenance. Latrine whose slabs and superstructures are well-maintained promotes its utilization by ensuring the privacy and safety of the users.

The distance from the health institution is inversely associated with latrine utilization in this study. When the household distance from the health institution increases, latrine utilization decreases. In another way, the odds of latrine utilization for near and medium households from the kebele office were 6.5 and 4 times more likely than those too far from the kebele office respectively. This is supported by another study conducted in Awabel District, Northwest Ethiopia. The households near woreda health center had more chance of latrine utilization (46). This might be attributed to the accessibility of health institutions and kebele offices that in turn could expose the households to information, follow-up, and latrine utilization.

Households whose latrine is found within ten (10) meters distance from the dwelling had 2.1 times increased odds of latrine utilization as compared to their counterparts. A consistent result was reported from the study in Awabel District, Northwest Ethiopia (46). This is because a distant latrine location can reduce the chance of utilization.

Unexpectedly, there is no significant difference in latrine utilization between CLTSH-implemented and non-implemented villages. This result is inconsistent with the finding of the studies in Laelai Maichew district and Hawassa town (37, 40). The possible reason for the discrepancy could be that currently CLTSH is not in active implementation in the current study area as it was in the initial time. The status of CLTSH implementation described in this study is ever implementation of CLTSH not necessarily the current implementation of CLTSH as this approach is almost failed the current context.

The strengths of this study are that latrine utilization and the associated factors are separately determined and compared between CLTSH-implementing and non-implementing villages. On top of this, it was a mixed method and tried to explore the CLTSH implementation status and the challenges faced in the implementation through qualitative data collection methods. However, it has its limitations. The first limitation is the cross-sectional nature of the study design which couldn't ascertain the direction of association between the independent factors and latrine utilization. Secondly, only two districts were involved which could affect the generalizability of the findings to whole districts in the East Wollega Zone.

Conclusion

The latrine utilization in this study was found to be lower as compared to many other studies in different parts of the country and the open defecation-free mobilization. Religion, educational status of the household head, occupation of the mother, absence of children <5 years, toilet cleaning, frequency of latrine construction, maintenance need of a toilet, distance from health institution, distance from kebele office, and latrine distance from dwelling were the factors that found to affect latrine utilization. Hence, households need to maintain their toilets on time, construct them proactively before they become out of service, clean their toilets frequently and make them ready for utilization at all times. Besides, better if they construct a latrine at an accessible distance from the dwelling in a manner that promotes its utilization and that does not contaminate the groundwater sources. In addition to this, the adults in the household have to demonstrate the latrine utilization for children, and safely dispose of the feces of small children who cannot use the toilet independently. Health workers and health officials including health extension workers should visit the households, teach them about toilet utilization and its advantage, and encourage them to appropriately utilize their toilets including the safe disposal of children's

feces. The local kebele leaders have to supervise the status of toilet construction, and utilization among the households of their catchment and urge them to prepare and utilize the prepared toilet effectively. Religious leaders should also encourage the construction and utilization of latrine as it prevents the chain of disease transmission and promotes the health of the society.

List of Abbreviations

CLTS: Community-led total sanitation, CLTSH: Community-led total sanitation and hygiene, FSM: Faecal sludge management, NGOs: non-governmental organization, OD: open defecation, ODF: open defecation Free, SDG: Sustainable Development Goal, WASH: Water, Sanitation and Hygiene

Ethical Considerations

This research was done according to the Helsinki declaration regarding the research ethical issues. Ethical clearance was obtained from the Wollega University, research ethics review committee before data collection with the reference number IHSRPTTAD90/2014. Institute of Health Sciences wrote the official letter to the East Wollega health department. Also, letter of cooperation was taken from the zonal health department and oral permission was obtained from the district Health offices before the start of data collection. All necessary measures were made to guard against any form of harm and discomfort to the study subjects. The study purpose, risks, and benefits were explained in the local language for the participants. Their informed, voluntary, written and signed consent, in the end, was sought and participants thumb printed consent to participate in the study. Confidentiality was also guarded by making sure that the study participants will not be represented by their names. In addition, password protection of soft data and the use of a key and lock for hard copy data was employed to guarantee confidentiality.

Consent for publication: not applicable

Availability of data and materials: The datasets used and/or analyzed during the current study are available from the corresponding author upon reasonable request.

Competing interests: The authors declare that they have no competing interests.

Funding: Funding not obtained.

Authors' contributions

ATS conceptualized the study, wrote the proposal, analyzed the data, wrote the report, and led the manuscript writing. DRT designed the methods and revised the proposal. AE and ETG engaged in

the design of the study and quantitative data analysis. MCC, JWF, and DSG designed the qualitative data collection methods and revised the study proposal. BB, BRF, and ML analyzed the data and prepared the manuscript. All authors read and approved the final manuscript.

Acknowledgment

Our thanks go to Wollega University for giving us this opportunity to conduct scientific research on the problems of the community. We would like to extend the special thanks to the East Wollega zonal health department, health offices in the study districts, and primary health care unit directors for giving us valuable information and permission to conduct the study. Finally, we thank the data collectors, supervisors, and study participants for their cooperation.

List of References

1. Mitchell C, Abeysuriya K, Ross K. Making pathogen hazards visible: a new heuristic to improve sanitation investment efficacy. Waterlines. 2016;35, 163-181. doi:10.3362/1756-3488.2016.014.
2. Peal A, Evans B, Blackett I, Hawkins P, Heymans C. Fecal sludge management (FSM): analytical tools for assessing FSM in cities. J Water, Sanit Hyg Dev. 2014;4, 371-383. doi:10.2166/washdev.2014.139.
3. WAITHAKA RW. LATRINE USE AND ASSOCIATED FACTORS AMONG RURAL COMMUNITY MEMBERS IN SAMBURU EAST SUB-COUNTY, SAMBURU COUNTY, KENYA. 2015. A THESIS SUBMITTED IN PARTIAL FULFILMENT OF THE REQUIREMENTS FOR THE AWARD OF THE DEGREE OF MASTER OF PUBLIC HEALTH IN THE SCHOOL OF PUBLIC HEALTH, KENYATTA UNIVERSITY.
4. Tadele T. Prevalence of home delivery. Int J Environ Study. 2014;28: 12-48.
5. WHO (World health Organization). Water, Sanitation and Hygiene for Accelerating and Sustaining Progress on Neglected Tropical Diseases. A Global Strategy 2015-2020. Available on ______Accessed on 22-9-2021.
6. Stocks ME, Ogden S, Haddad D, Addiss DG, McGuire C, Freeman MC. Effect of water, sanitation, and hygiene on the prevention of trachoma: a systematic review and meta-analysis. PLoS Med. 2014;11(2):e1001605. doi: 10.1371/journal.pmed.1001605. eCollection 2014 Feb.
7. Beyene A, Hailu T, Faris K, Kloos H. Current state and trends of access to sanitation in Ethiopia and the need to revise indicators to monitor progress in the Post-2015 era. BMC Public Health. 2015;15:451. DOI 10.1186/s12889-015-1804-4.
8. Degebasa MZ, Weldemichael DZ, Marama MT. Diarrheal status and associated factors in under-five years old children in relation to implemented and unimplemented community-led total sanitation and hygiene in Yaya Gulele in 2017. . Pediatr Heal Med Ther. 2018;9:109-21.
9. Gebremariam B, Hagos G, M. A. Assessment of community led total sanitation and hygiene approach on improvement of latrine utilization in Laelay Maichew District, North Ethiopia. A comparative cross-sectional study. . PLoS One. 2018;13:1-11.
10. Venkataramanan V, Crocker J, Karon A, J. B. Community-led total sanitation: A mixed-methods systematic review of evidence and its quality. Environ Health Perspect. 2018;126:026001.
11. Tessema RA. Assessment of the implementation of community-led total sanitation, hygiene, and associated factors in Diretiyara district, Eastern Ethiopia. PLoS One. 2017;12:1-11.
12. Bain R, Cronk R, Wright J, Yang H, Slaymaker T, Bartram J. Fecal contamination of drinking-water in low- and middle-income countries: a systematic review and meta-analysis. PLoS Med. 2014;11(5):e1001644.

13. Plan International Ethiopia. Trainers' Guide on Community-Led Total Sanitation and Hygiene (CLTSH) Approaches. September, 2012.
14. Robert Ch. Going to scale with community-led total sanitation: Reflections on experience, issues and ways forward: Institute of Development Studies practice paper. 2009;1: 10p.
15. Araya M. UNICEF Ethiopia. Leadership matters: the case of community led total sanitation and hygiene, Unite for children of Ethiopia, April 13, 2016.
16. Saha S, Negussie A. Community-led total sanitation (CLTS): Addressing the challenges of scale and sustainability in rural Africa. Desalination. 2009;000: 1-8. 2009 ELSEVIER B.V.
17. Andres HG. Pathways to sustainability in Community-Led Total Sanitation: Experiences from Madhya Pradesh and Himachal Pradesh. Polytechnic University of Valencia, 2013. ISBN number: 978-84-9048-112-7. [Cited 2021 August 29] available: https://riunet.upv.es.
18. David D., Macharia K. United Nations Summit for the adoption of the Post-2015 development agenda, The 2030 Agenda for Sustainable Development, 2015 [Cited 2021 August 29] Available: http://wwwunorg/pga/wp-ontent/uploads/sites/3/2015/08/120815_outcome-document-of-Summit-for-adoption-ofthe-post-2015-development-agendapdf.
19. UNICEF; WHO. 25 years progress on sanitation and drinking water: 2015 update and MDG assessment, Geneva, Switzerland, 2015, 4p-5p. ISBN number 9789241 509145.[Cited 2021 Aug. 29] Available: http://www.who.int/water_sanitationhealth/publications/jmp-2015-update/en/.
20. Ethiopian Public Health Institute (EPHI) [Ethiopia] and ICF. 2021. *Ethiopia Mini Demographic and Health Survey 2019: Final Report*. Rockville, Maryland, USA: EPHI and ICF.
21. Abebe TA, Tucho GT. Open defecation-free slippage and its associated factors in Ethiopia: a systematic review. systematic reviews. 2020; 9:252; https://doi.org/10.1186/s13643-020-01511-6.
22. Leshargie CT, Alebel A, Negesse A, Mengistu G, Wondemagegn AT, Mulugeta H, et al. Household latrine utilization and its association with educational status of household heads in Ethiopia: a systematic review and meta-analysis. BMC Public Health. 2018;18:901: https://doi.org/10.1186/s12889-018-5798-6.
23. Zewdie A, Dugassa W, Mannekulih E, Kaba Z, Wondimu R. Latrine Utilization and Associated Factors Among Households in SebetaHawas Woreda, Oromia Special Zone, Ethiopia European Journal of Clinical and Biomedical Sciences. 2021;Vol. 7, No. 2, pp. 44-52. doi: 10.11648/j.ejcbs.20210703.12.
24. Abebe AM, Kassaw MW, Mekuria AD, Yehualshet SS, Fenta EA. Latrine Utilization and Associated Factors in Mehal Meda Town in North Shewa Zone, Amhara Region, Ethiopia, 2019. BioMed Research International. 2020;https://doi.org/10.1155/2020/7310925.
25. Yimam YT, Gelaye KA, Chercos DH. Latrine utilization and associated factors among people living in rural areas of Denbia district, Northwest Ethiopia, 2013, a cross-sectional study. Pan African Medical Journal 2014; 18:334 doi:1011604/pamj2014183344206.
26. Koyra HC, Sorato MM, Unasho YS, Kanche ZZ. Latrine Utilization and Associated Factors in Rural Community of Chencha District, Southern Ethiopia: A Community Based Cross-Sectional Study. *American Journal of Public Health Research*. 2017;vol. 5, no. 4; 98-104. doi: 10.12691/ajphr-5-4-2.
27. Ayalew D, Boelee E, Endeshaw T, Petros B. Cryptosporidium and Giardia infection and drinking water sources among children in Lege Dini, Ethiopia. Tropical Medicine and International Health. 2008;13(4): 472-475. : https://doi.org/10.1111/j.365-3156.2008.02024.x PMID: 18282239.
28. Federal Democratic Republic of Ethiopia Ministry of Health (FDREMOH). National drinking water quality monitoring and surveillance strategy, Addis Ababa, May 2011. 10p.
29. Thewodros B, Seyoum L. Water Supply and Health: Drinking water and Sanitation Coverage in Ethiopia 1990–2015 Review. International Journal of Environment, Agriculture and Biotechnology (IJEAB). 2016;1:1.

30. Kedir AA. Prevalence of diarrheal disease in under five children and associated risk factors in wolitta soddo town, southern, Ethiopia. ABC Research Alert. 2015;3:2: [Cited 2021, August 20] Published Online, Available: http://abcreal.weebly.com/.
31. Nucleus Research P.L.C. African Medical and Research Foundation in Ethiopia: Baseline survey report: Access to water, sanitation and hygiene promotion project, Gullele sub city, district 05, Addis Ababa, Ethiopia; 2011.

32. Mengistie B, Baraki N. Community based assessment on household management of waste and hygiene practices in Kersa Woreda, Eastern Ethiopia. Ethiop J Health Dev. 2010;24: 103-109.
33. Heijnen MCO, Peletz R. Shared sanitation versus individual household latrines: A systematic review of health outcomes. PLoS ONE. 2014;9: 4.
34. Awoke W, Muche SA. Latrine coverage and associated factors among rural communities in the District of Bahir Dar Zuria, Ethiopia: Cross sectional study. BMC Public Health. 2013;13: 1471-2458.
35. WSP (Water safety plan). Desk review on Economics of Sanitation (ESI) for Ethiopia. 2015; unpublished.
36. East Wollega Zonal health department Annual performance Review. August 2021, Farmland Hotel, Nekemte, Oromia, Ethiopia.
37. Gebremariam B, Tsehaye K. Effect of community led total sanitation and hygiene (CLTSH) implementation program on latrine utilization among adult villagers of North Ethiopia: a cross-sectional study. BMC Research Notes. 2019; 12:478; https://doi.org/10.1186/s13104-019-4519-2.
38. World Health Organization and United nations children's fund 2006. CORE QUESTIONS ON DRINKING-WATER AND SANITATION FOR HOUSEHOLD SURVEYS. Available from World Health Organization Water, Sanitation and Health 20, Avenue Appia, CH-1211 Geneva 27, Switzerland http://www.who.int/water_sanitation_health or Water, Environment and Sanitation Section 3 United Nations Plaza, New York, NY 10017, USA. http://www.unicef.org/wes. Accessed on 27/8/2021.
39. Asnake D, Adane M. Household latrine utilization and associated factors in semiurban areas of northeastern Ethiopia. PLoS ONE. 2020;15 (11): e0241270. https://doi.org/10.1371/journal. pone.0241270.
40. Tulu L, Kumie A, Hawas SB, Demissie HF, Segni MT. Latrine utilization and associated factors among kebeles implementing and non implementing Urban Community Led Total Sanitation and Hygiene in Hawassa town, Ethiopia. African Journal of Environmental Science and Technology. 2017;11(3),151-162, DOI: 10.5897/AJEST2016.2223.
41. Anteneh A, Kumie A. Assessment of the impact of latrine utilization on diarrhoeal diseases in the rural community of Hulet Ejju Enessie Woreda, East Gojjam Zone, Amhara Region. *Ethiop J Health Dev*. 2010;24(2);110-118.
42. Gebremedhin G, Tetemke D, Gebremedhin M, Kahsay G, Zelalem H, Syum H, et al. Factors associated with latrine utilization among model and non-model families in Laelai Maichew Woreda, Aksum, Tigray, Ethiopia: comparative community based study. BMC Res Notes. 2018;11:586; https://doi.org/10.1186/s13104-018-3683-0.
43. Gebremedhin H, Abay T, Gebregzabher T, Yemane D, Gebreegziabiher G, Belay S. LATRINE UTILIZATION AND ASSOCIATED FACTORS IN SOUTH EAST ZONE OF TIGRAY REGION, NORTH ETHIOPIA. European Journal of Biomedical and pharmaceutical sciences. 2016;Volume 3, Issue 6, 120-126.
44. Ashenafi T, Dadi AF, Gizaw Z. Latrine utilization and associated factors among Kebeles declared open defecation free in Wondo Genet district, South Ethiopia, 2015. ISAAB J Health Environ Sci. 2018;Vol. 5(5), pp. 43-51, DOI: 10.5897/ISAAB-JHE2018.0050.
45. Woyessa ET, Ashenafi T, Ashuro Z, Ejeso A. Latrine Utilization and Associated Factors Among Community-Led Total Sanitation and Hygiene (CLTSH) Implemented Kebeles in Gurage Zone, Southern Ethiopia: A Cross-Sectional Study. Environmental Health Insights. 2022;16: 1–10.

46. Gedefaw M, Amsalu Y, Tarekegn M, Awoke W. Opportunities, and Challenges of Latrine Utilization among Rural Communities of Awabel District, Northwest Ethiopia, 2014. *Open Journal of Epidemiology*. 2015;**5**: 98-106. http://dx.doi.org/10.4236/ojepi.2015.52013.

Click here to access/download;Figure;Fig 1.tiff

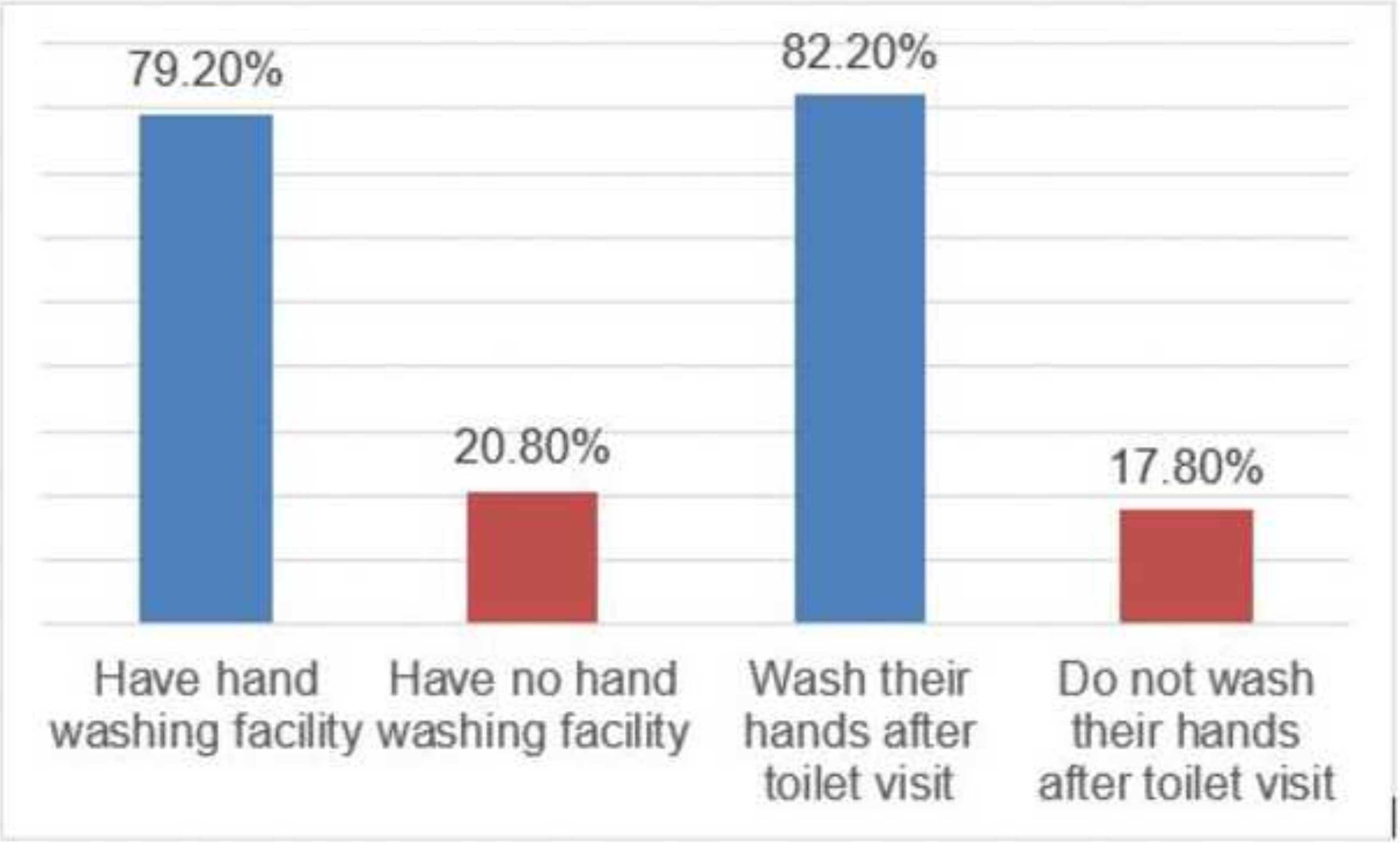

Click here to access/download;Figure;Fig 2.tiff

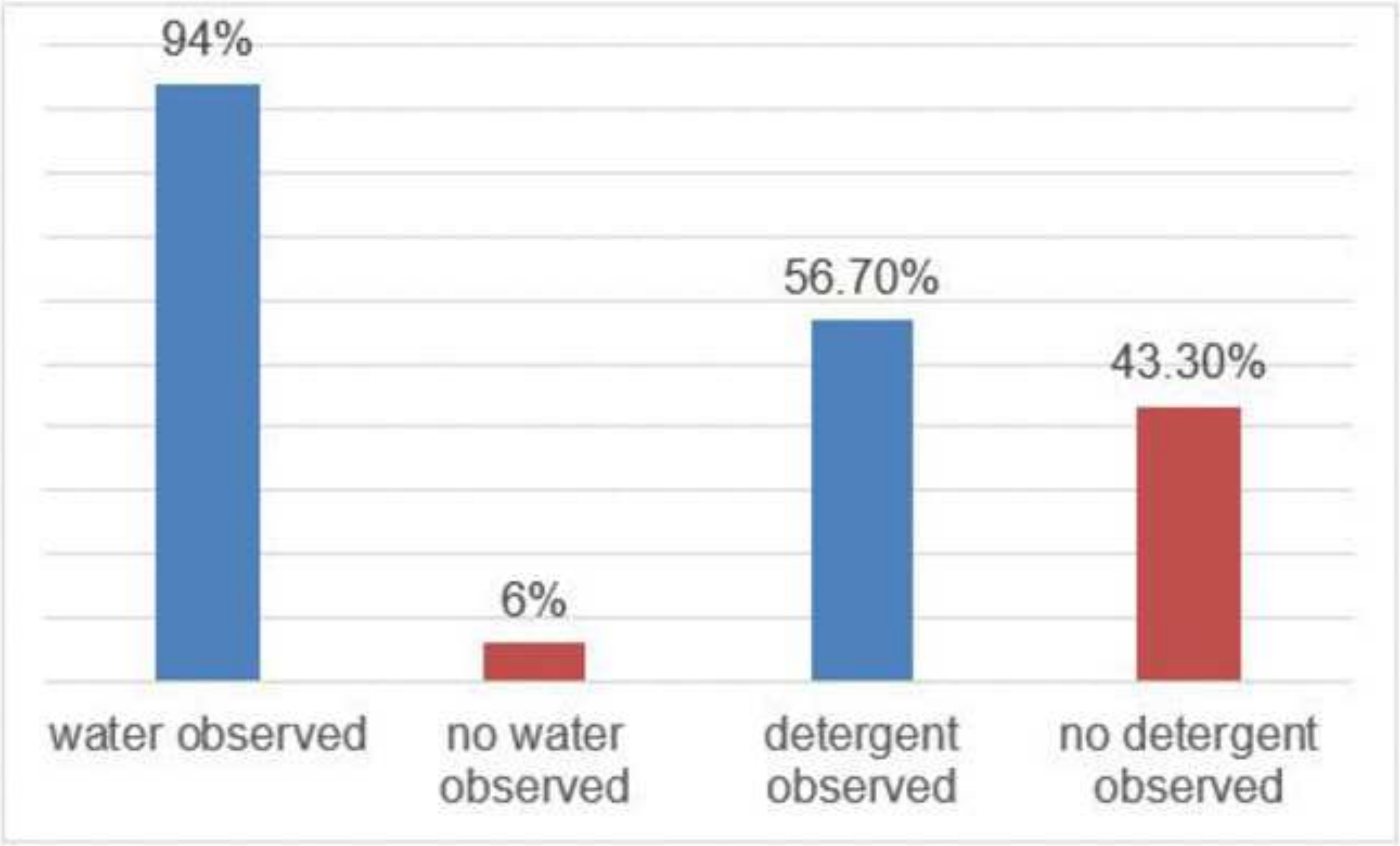

Click here to access/download;Figure;Fig 3.tiff

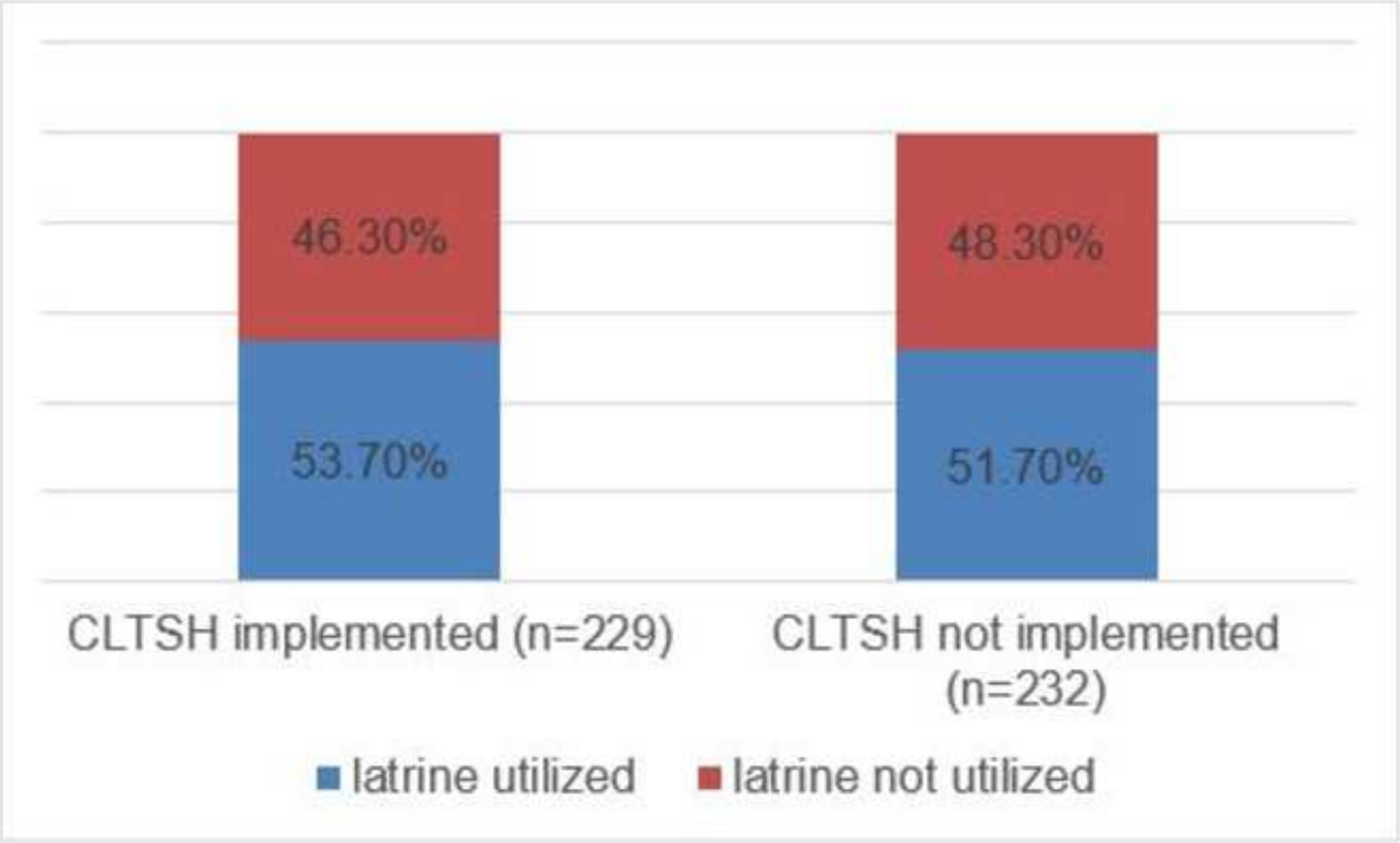

Click here to access/download;Figure;Fig 4.tiff

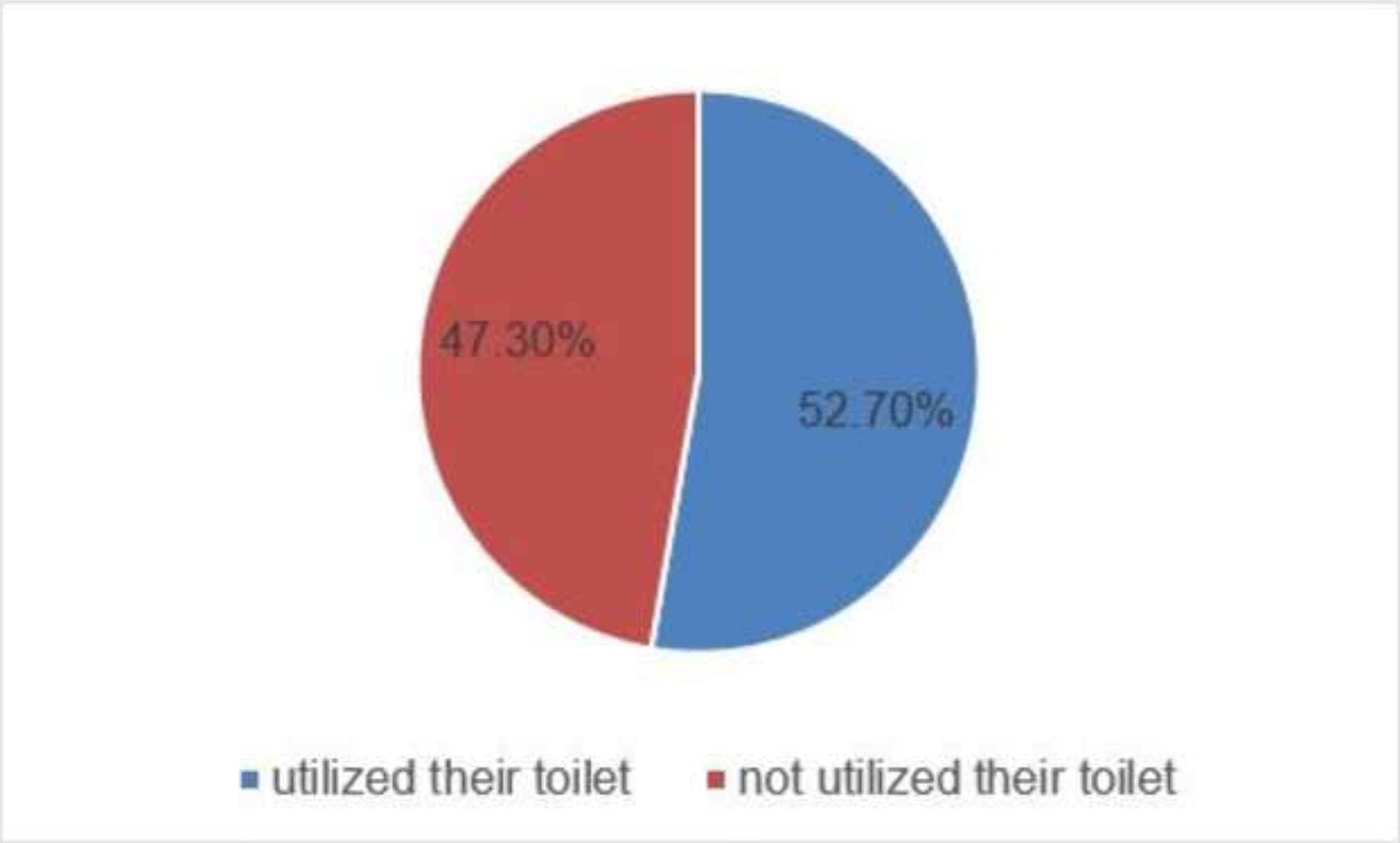

supporting information

Click here to access/download
Supporting Information
STROBE-checklist-v4-combined-PlosMedicine.docx
