# Supplementary material for: Latrine utilization and associated factors among districts implementing and not-implementing community-led total sanitation and hygiene in East Wollega, Western Ethiopia: A comparative cross-sectional study
Source: PLoS One. 2023 Jul 13;18(7):e0288444. doi: 10.1371/journal.pone.0288444 (PMC10343159; doi:10.1371/journal.pone.0288444)
Supplement: Attachment — Submitted filename: Responses to reviewers.docx [file pone.0288444.s004.docx]

**Authors’ point-by-point response to the editor and Reviewers comments**

First, the authors would like to thank the editor and reviewers for reviewing our manuscript and providing us the constructive comments that help us to enrich the document. We tried to address all the comments and made necessary modifications and clarifications based on the comments and suggestions provided.

**Comments by editor**

1. Please ensure that your manuscript meets PLOS ONE's style requirements, including those for file naming. The PLOS ONE style templates can be found at

<https://journals.plos.org/plosone/s/file?id=wjVg/PLOSOne_formatting_sample_main_body.pdf> and

<https://journals.plos.org/plosone/s/file?id=ba62/PLOSOne_formatting_sample_title_authors_affiliations.pdf>.

We opened those links and checked the alignment of our manuscript with the formats and styles required.

2. We note that you have indicated that data from this study are available upon request. PLOS only allows data to be available upon request if there are legal or ethical restrictions on sharing data publicly. For more information on unacceptable data access restrictions, please see <http://journals.plos.org/plosone/s/data-availability#loc-unacceptable-data-access-restrictions>.

In your revised cover letter, please address the following prompts:

a) If there are ethical or legal restrictions on sharing a de-identified data set, please explain them in detail (e.g., data contain potentially sensitive information, data are owned by a third-party organization, etc.) and who has imposed them (e.g., an ethics committee). Please also provide contact information for a data access committee, ethics committee, or other institutional body to which data requests may be sent.

b) If there are no restrictions, please upload the minimal anonymized data set necessary to replicate your study findings as either Supporting Information files or to a stable, public repository and provide us with the relevant URLs, DOIs, or accession numbers. For a list of acceptable repositories, please see <http://journals.plos.org/plosone/s/data-availability#loc-recommended-repositories>.

We will update your Data Availability statement on your behalf to reflect the information you provide.

The data set is uploaded

3. Your ethics statement should only appear in the Methods section of your manuscript. If your ethics statement is written in any section besides the Methods, please delete it from any other section.

Accepted.

4. We note that you have referenced (WSP (Water safety plan). Desk review on Economics of Sanitation (ESI) for Ethiopia. 2015; unpublished.]) which has currently not yet been accepted for publication. Please remove this from your References and amend this to state in the body of your manuscript: as detailed online in our guide for authors

<http://journals.plos.org/plosone/s/submission-guidelines#loc-reference-style> "

Corrected

5. Review Comments to the Author

Abstract
Comment 1: you didn’t provide a good justification to conduct the study under the background. Amended in this revised manuscript.
Comment 2: the result is not written in concise and clear manner as well very congested and doesn’t attract the reader. Amended.
Background
Comment 1: in the last paragraph of the background you had tried to justify the need for the study and tried to show some strengths of the study. In the paragraph you have mentioned that other studies didn’t investigate safely managed services and this study fills the gap. However, in this study nothing has been showed in the methods (especially operational definition) and result (Latrine coverage by sanitation ladder) about safely managed services. So, you better exclude this justification otherwise include the above comments in your methods and result part.

We accepted this suggestion and removed the description about ‘safely managed services’ from the manuscript.

Methods and materials
Comment 1: for the qualitative study you planned and executed thematic analysis. However your result didn’t show that and the qualitative result is not presented in a good manner. As well the discussion doesn’t incorporate findings from the qualitative study. By the way, it is better to exclude the qualitative study whole, if not so try to read and read about triangulation and thematic analysis and come up with an organized result and discussion.

We accepted this and removed the qualitative part.

Comment 2: there is no need to have these paragraphs in the operational definition “The use of the latrine was assessed based on self-reporting, and the observation of proxy indicators. Accordingly” and “Finally, the household was categorized as utilized or not utilized based on the above definition”

Amended.

Comment 3: as per the operational definition Latrine utilization is measured among households having an improved toilet. So what are improved latrines/toilets? ; operationalize the facilities and try to show the reader how many of the households own improved toilets in your manuscript clearly. Furthermore what is a functional toilet? This also needs to be operationalized.

Accepted and operationalized in this revised manuscript..

Comment 4:
Result
Comment 1: almost all the tables are distorted and poorly organized; this needs to be clearly addressed.

The tables are modified

Comment 2: what is the purpose of having this much category of age groups? I think you better categorize age not more than 4 or 5 age groups.

We recategorized the age based on the reviewer’s suggestion.

Comment 3: line 278 to 280, what is Squat hole cover? ; What you are talking about is a slab not a squat hole cover.

Thank you. We corrected the variable as “presence of slabs for the latrine”

Comment4: line 290 water, sanitation and hygiene focal person better be “WaSH focal person”

Comment 5: for the qualitative study, all the second person paragraphs or ideas better be italic and should be placed separately.

These comments were addressed as we already removed the qualitative part from this manuscript.

Comment 6: line 297 and 302, A 34 what? How
Comment 7: the variable distance from the kebele. How near is near and how far is far? This should have to be operationalize.

Thank you! We specified this as near if walking distance of <30 minutes, medium (30-60 minutes), and too far (>60 minutes).
Comment 8: what does the variable frequency of latrine construction means? We tried to make this variable clear by modifying as “number of times latrines constructed since household establishment”
Comment 9: the quantitative data focuses on latrine utilization and the qualitative result showed about issues other than that. Therefore as I tried to mention earlier it would be better to exclude from the manuscript. We accepted this comment.
Discussion
Comment 1: generally almost all your justifications are not supported by evidences from other literatures. You just forward your personal opinion all over the discussion. So please try to read and incorporate other literatures.

We accepted this comment, modified the discussion and supported our justification by references.

Reviewer #2: The manuscript was well written and only have minor comments. The problem is stated clearly, and the result written in well manner. There was not too much grammatical error in the manuscript and all data incorporated in the result part.

Thank you! I hope those minor comments have been addressed in this revised version.
